# Impact of a Momentary Mindfulness Intervention on Rumination, Negative Affect, and their Dynamics in Daily Life

**DOI:** 10.1007/s42761-024-00291-9

**Published:** 2025-01-24

**Authors:** Teresa Bolzenkötter, Andreas B. Neubauer, Peter Koval

**Affiliations:** 1 Freie Universität Berlin, Berlin, Germany; 2 RWTH Aachen University, Aachen, Germany; 3 University of Melbourne, Melbourne, Australia

**Keywords:** Mindfulness, Rumination, Negative affect, Micro-randomized trial, Ambulatory assessment

## Abstract

Rumination and negative affect are mutually reinforcing experiences. Their dynamic relation can confer vulnerability to psychopathology. Cultivating mindfulness has been proposed to buffer against such downward spirals of negativity. However, it remains unclear whether practicing mindfulness in daily life causally impacts rumination, negative affect, and their dynamics. We investigated this using a micro-randomized intensive longitudinal trial. Participants (*N* = 91) were prompted eight times per day for 10 days using a smartphone app. At each prompt, participants were randomized to complete a brief mindfulness intervention or an active-control task and then reported levels of rumination and negative affect. Results of dynamic structural equation models showed that the mindfulness intervention led to lower levels of rumination and negative affect but that it had no reliable impact on their dynamics. Thus, cultivating mindfulness in daily life may be a promising approach for decreasing rumination and negative affect but not their dynamical relation.

**Supplementary Information:**

The online version contains supplementary material available at 10.1007/s42761-024-00291-9.

Rumination is a common form of repetitive negative thinking involving persistent distressing thoughts (Martin & Tesser, 2006; Nolen-Hoeksema et al., 2008). Not only is rumination unpleasant, it is theorized to serve as a transdiagnostic risk factor for mental disorders, such as depression (Ehring & Watkins, 2008; Kircanski et al., 2015; McEvoy et al., 2013). One key mechanism through which rumination may confer vulnerability to psychopathology is via its dynamical association with negative affect (NA). That is, rumination increases unpleasant feelings (e.g., sadness, anxiety, anger) which, in turn, predict more ruminative thinking (e.g., Blanke et al., 2022; Moberly & Watkins, 2008), leading to a mutually reinforcing cycle (Garland et al., 2010; Selby et al., 2016). From a dynamical systems perspective, stronger reciprocal associations among rumination and NA may amplify the impact of stressors, such that even relatively mild events can have considerable and persistent psychological effects, potentially hastening an individual’s transition into psychopathology (e.g., Wichers et al., 2015). Consistent with this, Stefanovic et al. (2021) found that individuals who showed a stronger association between rumination and NA in daily life were at higher risk of developing depressive symptoms over the following three months. Moreover, rumination and NA each have their own temporal persistence, or *inertia*, independent of their reciprocal associations (Bean et al., 2020; Blanke et al., 2022). Higher inertia of rumination (Bean et al., 2020, 2021) and NA (Houben et al., 2015) have also both been linked with psychopathology. Thus, the mutually and self-reinforcing dynamics of rumination and NA may have important mental health implications, making them an important target for interventions.

Mindfulness-based interventions, which typically involve the cultivation of purposeful, curious, non-judgmental, or non-reactive momentary awareness (Kabat-Zinn & Hanh, 2009; Van Dam et al., 2018), are promising candidates for reducing rumination and negative affect and for interrupting the reinforcing dynamics among rumination and NA. Mindfulness is thought to exert its beneficial effects by cultivating non-judgmental awareness of one’s cognitive and affective experiences (Segal et al., 2002). This, in turn, is theorized to reduce the likelihood of automatic and potentially detrimental reactions to one’s experiences, such as rumination, which is known to prolong and intensify negative affect (Segal et al., 2002). Thus, by helping to decouple negative feelings/thoughts from perseverative meta-cognitions (e.g., thinking “why do I always react this way?”), mindfulness may reduce the reciprocal relation between negative affect and rumination.

Evidence that mindfulness may reduce rumination, NA, and their reciprocal association, has been observed both in the lab and in daily life. For example, a meta-analysis of lab experiments showed that inducing state mindfulness reliably reduced rumination and NA (Leyland et al., 2019). Well-controlled lab experiments permit strong causal inferences about the effects of mindfulness on cognitive and affective processes. However, experiments often lack ecological validity, making it unclear whether effects observed in the lab generalize to daily life.

Ambulatory assessment, including the experience sampling method (ESM), allows researchers to investigate how mindfulness influences spontaneous thoughts and feelings in daily life. Previous ESM studies have found that mindfulness was associated with lower levels of rumination and NA, either by measuring natural fluctuations in state mindfulness (Blanke et al., 2018, 2020; Brown & Ryan, 2003), or randomizing individuals to a mindfulness versus control intervention (Bolzenkötter et al., 2024; Rowland et al., 2020). Moreover, a few studies suggest that mindfulness might also moderate the temporal dynamics of these experiences. For example, Blanke et al. (2020) reported that the within-person association between rumination and NA was attenuated at times when people reported higher levels of mindfulness in daily life. Other studies have also linked trait mindfulness with lower inertia of NA (Keng & Tong, 2016; Rowland et al., 2020). In sum, a handful of ESM studies have demonstrated that mindfulness may influence levels and temporal dynamics of rumination and NA in daily life.

However, there is still a dearth of studies experimentally manipulating mindfulness and assessing its effects on immediate experiences and their dynamics in daily life. It thus remains unclear whether cultivating mindful states could lead to less unpleasant thoughts and feelings in the moment and/or interrupt their mutually and self-reinforcing dynamics. Importantly, no previous studies have investigated the within-person causal effects of mindfulness on rumination and NA in daily life.

The within-person effect of an intervention represents the degree to which an individual reports more or less of an outcome on occasions when they are (vs. are not) exposed to that intervention. From a theoretical perspective, within-person effects directly operationalize how psychological processes are hypothesized to unfold over time, within individuals, following changes to a person’s internal/external environment (Bolger & Laurenceau, 2013). In contrast, between-person effects in traditional randomized controlled trials (RCTs) compare an outcome among different individuals randomized to a treatment vs. a control condition. Although traditional RCT designs can be used to estimate the average (within-person) causal effect of an intervention across individuals, this only applies “in aggregate” or under the (rather unrealistic) assumption that the causal effect is identical for all individuals (Rohrer & Murayama, 2023). Importantly, within-person effects are not necessarily identical to between-person effects (Molenaar, 2004) and the extent to which a within-person effect will translate into a corresponding between-person effect depends on various factors that need to be empirically investigated (Neubauer et al., 2024).

## The Present Study

We aimed to fill this gap by investigating the within-person causal effects of a brief momentary mindfulness intervention on rumination, NA, and their dynamics in daily life. We analyzed data from the Momentary Mindfulness and Everyday Emotion (MMEE) study, in which participants were randomized to complete either a mindfulness intervention or an active-control task eight times per day for 10 days, using a smartphone app. Unlike a traditional RCT, where participants are randomized to an intervention or control group, the MMEE study adopted a micro-randomized design, in which each participant received both intervention and control at different occasions (Klasnja et al., 2015; Qian et al., 2022). In such designs, each person serves as their own control, allowing us to estimate within-person causal effects of mindfulness on psychological experience. Immediately after completing the mindfulness intervention or active-control task, participants reported their levels of rumination and NA. Drawing on the literature reviewed above, we hypothesized that, relative to the active-control task, completing the mindfulness intervention would lead to (i) lower levels of rumination (H1) and NA (H2); (ii) weaker within-person effects of rumination on NA (H3) and NA on rumination (H4); and (iii) weaker temporal persistence (i.e., inertia) of rumination (H5) and NA (H6).

## Method

The MMEE study was approved by the University of Melbourne Human Research Ethics Committee (No. HREC No. 2056669.2). Participants completed the study in five batches (approximately 20–30 people per batch) between June 11th and August 22nd, 2020. Thus, data collection took place during the COVID-19 pandemic, when rumination and negative affect may have been particularly prevalent. All participants gave informed consent and were reimbursed up to £75.50, with reimbursement partly tied to ESM compliance (for details, see https://osf.io/cdaxb).

### Participants

Our final sample comprised 91 Australian residents, whose mean age was 29.2 years (*SD* = 8.99) and of whom 51.6% were female (46.2% male, 2.2% other). Our sample can be considered a convenience sample: participants were recruited via the online research platform Prolific (www.prolific.co) and enrolled in a study investigating how brief mindfulness training impacts everyday emotions. To be eligible, participants were required to (1) be aged at least 18 years; (2) be fluent in English; (3) have a smartphone running Android or iOS; (4) have normal or corrected-to-normal vision; (5) have no hearing loss/difficulties; and (6) have no untreated mental health conditions impacting their daily functioning. See Supplemental Material for divergence of this inclusion criteria from the original pre-registration as well as for additional demographic characteristics.

After initially recruiting 146 participants, 28 participants did not commence the ESM phase (due to technical reasons or voluntarily withdrawal). Additionally, 21 participants whose compliance with the ESM protocol or the intervention was low were excluded during data collection (for detailed exclusion criteria, see https://osf.io/cdaxb). Finally, after inspecting the data, we excluded six more participants with low intervention compliance who had not been previously excluded during data collection. We report supplemental analyses using all available data, including from these six participants, which support identical conclusions. We retained data from two additional participants with low ESM compliance who were not excluded during data collection. Flow of participants is included in Figure S1 in the Supplemental Material.

#### Sample Size and Power

Based on Schultzberg and Muthen’s (2018) guidelines for power in DSEM, we originally aimed to recruit a sample of *N* = 150 participants sampled at *T* = 80 occasions to obtain a sufficiently large sample even after participant attrition, exclusions, and missing data. Due to time and funding constraints, we stopped data collection after recruiting 146 participants. After exclusions and attrition (described above), our final sample size comprised *N* = 91 participants each with approximately *T* = 63 complete ESM surveys. Given that this is likely underpowered for detecting between-person and/or cross-level interaction effects (Schultzberg & Muthén, 2018), we decided to focus exclusively on within-person effects in the current report. Schultzberg and Muthen’s (2018) findings indicate that within-person parameters can be estimated with low relative bias and good coverage with *N* and *T* between 50 and 75, which our final sample fulfills. Finally, we ran a power analysis using Murayama et al.’s (2022) online multilevel power calculator to estimate whether our final sample was sufficient to detect within-person interaction effects of similar magnitude as reported by Blanke et al. (2020). This analysis indicated that a Level-2 sample of *N* = 90 would be sufficient to yield 80% power of detecting a within-person effect equivalent to the smallest significant interaction effect observed by Blanke et al. (2020) (i.e., Study 1: rumination x mindful-attention predicting NA: *Est*. = –.05, *SE* = .02, *t* = –2.5, Level-2 *N* = 70). Thus, our final sample size (*N* = 91) was adequately powered to detect within-person effects of similar magnitude as those reported by Blanke et al. (2020).

### Experimental Design

This study adopted a novel cross-classified experimental design, whereby the mindfulness intervention was randomized at each occasion (within persons), whereas the probability of receiving the intervention (vs. active-control task) was randomly assigned between persons *and* between occasions. We adopted this design to allow estimation of within-person, between-person, and between-occasion causal effects. However, as preliminary analyses indicated negligible between-occasion variance in our outcomes (see Supplemental Material), we opted to estimate two-level (rather than cross-classified) models. Moreover, as discussed above, given our final sample comprised *N* = 91 participants, we were likely underpowered to detect between-person effects and thus our analyses focus exclusively on within-person effects. However, our analytic approach (detailed below) accounts for between-person differences by modeling random effects for all within-person parameters. For more detail about the study design and a discussion of statistical approaches for estimating causal effects using this design, please see Neubauer et al. (2024).

## Materials and Procedure

### Baseline Session

Two days prior to the ESM phase, participants completed an online baseline session, during which they provided informed consent, reported basic demographic information (e.g., age, gender, education, ethnicity), and their previous meditation experience and practice frequency (see Table 1). Our sample can be considered relatively young and highly educated. Additionally, they completed several validated retrospective questionnaires that were not analyzed as part of this study (see https://osf.io/cdaxb).

**Table 1:** Demographic information as well as experience and current practice of meditation

Age in years (*M*, *SD*)		29.2 (8.99)
Gender (*n*, %)		
	female	47 (51.6)
	male	42 (46.2)
	other	2 (2.2)
Ethnicity (*n*, %)		
	Caucasian	60 (65.9)
	Asian	25 (27.5)
	Aboriginal or Torres Strait Islander	0 (0)
	African or African American	1 (1.1)
	Middle Eastern	1 (1.1)
	Hispanic	0 (0)
	other	4 (4.4)
Education (*n*, %)		
	did not complete high school	0 (0)
	high school	19 (20.9)
	trade, technical or vocational training	11 (12.1)
	bachelor degree	43 (47.3)
	postgraduate degree	18 (19.8)
Experience with meditation (*n*, %)		
	I have practiced meditation for a year or longer	2 (2.2)
	I have practiced meditation for 1–12 months	4 (4.4)
	I have practiced meditation for 1–4 weeks	6 (6.6)
	I have tried meditation a few times	56 (61.5)
	I have no previous experience with meditation	23 (25.3)
Practicing meditation (*n*, %)		
	no	70 (76.9)
	yes, occasionally	16 (17.6)
	yes, weekly	3 (3.3)
	yes, daily or more	2 (2.2)

*M* mean, *SD* standard deviation, *n* number of participants, % percent of participants

### ESM Phase

One day prior to the ESM phase (i.e., day after baseline), participants installed the ESM smartphone app, *SEMA3* (O’Brien et al., 2024), and watched videos with detailed instructions about how to use SEMA3, and explaining the content of the ESM survey as well as the mindfulness intervention and active-control tasks. That evening, participants received two practice ESM surveys, which were excluded from analyses.

The ESM phase began the next day. Over the following 10 days, participants received eight ESM surveys per day scheduled between 9:00 a.m. and 8:40 p.m. following a stratified random-interval scheme (approximately one survey every 90 min). ESM surveys expired after 40 min to ensure no overlap between successive surveys (see https://osf.io/cdaxb for more detail on the ESM protocol).

#### Mindfulness Intervention and Active-Control Task

At each ESM survey, participants were randomly assigned to complete either a mindfulness intervention or an active-control task, which each involved listening to a short audio track hosted on Soundcloud.com. For the mindfulness intervention (https://soundcloud.com/momentary-mindfulness/task-one), we used a freely available recording of Williams and Penman’s (2011) “Three Minute Breathing Space”, an audio-guided mindfulness meditation exercise lasting 3 min and 22 s. The exercise invited participants to attend to their thoughts and feelings, and especially to the sensation of their breathing, with openness and curiosity. The active-control task (https://soundcloud.com/momentary-mindfulness/task-two) was an audio clip containing neutral background sound (ambient recordings of public places, such as cafes), which we edited to be approximately equivalent to the mindfulness intervention in terms of duration and audio profile (i.e., volume, number of silences, etc.). We included the active-control task to determine if any observed effects of the mindfulness intervention were simply due to the possible distracting effects of interrupting current thoughts/activities by listening to a short audio clip. After completing their assigned task, participants were asked to return to SEMA3 and complete several ESM items assessing momentary experiences of negative affect and rumination; these allowed us to investigate the acute impact of each brief mindfulness intervention.

#### ESM Items

ESM surveys included a total of 15 items, all rated on slider scales from 0 (*not at all*) to 100 (*very much*). Below, we describe the eight ESM items relevant to the current study (see https://osf.io/cdaxb for details of other ESM items). The first three ESM items assessed rumination and mindfulness experiences during the intervention (or active-control) task. These items were presented in a random order at each ESM survey.

#### Rumination

We assessed state rumination using the item “Over the last few minutes, did you find yourself getting stuck on your feelings and problems?” This item is similar to Kircanski et al.’s (2015) measure of state rumination. Our item differs from Kircanski et al.’s in terms of the time-frame (“over the last few minutes”) as well as the phrase “getting stuck on”, which we included to capture the core ruminative feature of uncontrollability (Rosenkranz et al., 2020).

#### Mindfulness

We assessed state mindfulness with two items adapted from the Toronto Mindfulness Scale (Lau et al., 2006) designed to capture the core mindfulness components of *curiosity* (“Over the last few minutes, were you curious about each thought/feeling that you had?”) and *decentering* (“Over the last few minutes, did you try to accept each thought/feeling you had, whether it was pleasant or unpleasant?”). For our manipulation check (see below), we combined the two mindfulness items into a state mindfulness composite (*r*_Within_ = .29; *r*_Between_ = .71).

#### Negative Affect

After completing the above rumination and mindfulness items, participants rated their momentary positive and negative feelings in response to the item “Right now, how [*adjective*] do you feel?” including five adjectives selected to capture high- and low-activation NA, as conceptualized in affective circumplex models (Yik et al., 2011): “sad”, “stressed”, “anxious”, “angry”, and “depressed”. We averaged each participant’s responses to the five negative items at each ESM survey to form an NA scale (ω_Within_ = .79; ω_Between_ = .94).

### Data Preparation

We prepared data using R version 4.0.5 (R Core Team, 2021). A technical error occurred on Day 1 of the ESM phase for the first batch of participants making these data unusable; we therefore removed these surveys prior to running analyses. Additionally, we considered ESM items completed in less than 750ms as potentially reflecting careless responding (McCabe et al., 2012) and replaced these with missing values. This affected 113 (.19%) of the 57,432 completed ESM items.

### Data Analyses

We analyzed data using dynamic structural equation modeling (DSEM; Asparouhov et al., 2018) implemented in Mplus 8.9 (Muthén & Muthén, 1998–2017). DSEM combines structural equation modeling with multilevel time-series modeling, making it ideally suited for analysis of intensive longitudinal data (Hamaker et al., 2023). DSEM has several benefits over standard multilevel regression as illustrated by McNeish and Hamaker (2020).

DSEM uses latent centering to decompose observed variables into between- and within-person components. The between-person component represents trait-like mean levels of observed variables, whereas the within-person component represents dynamic deviations of observed variables around their stable mean levels. The latter were of primary interest to us as our hypotheses concerned within-person dynamic effects. Consequently, we describe only the within-person parts of our DSEM models in detail below. We include full model diagrams depicting latent variable decomposition and within-, and between-person models in the Supplemental Material (see Figures S2-S10).

We ran two-level models to account for ESM surveys as nested within persons. All within-person parameters were modeled as random effects that could vary between persons. At the between-person level, all random effects were allowed to freely correlate (i.e., we estimated an unstructured covariance matrix). We used Bayesian estimation with Mplus's default uninformative priors and we checked model convergence using posterior scale reduction (PSR) values < 1.05 after 5000 iterations with a thinning factor of 10. When a model did not converge, we re-ran the model with double the iterations and again checked convergence. When a model converged, we also re-ran the model with double the iterations to check that convergence was stable. We report the results of all final models (i.e., with double the number of iterations required to achieve convergence). We considered effects as meaningfully different from zero (i.e., “significant”) when their 95% credible interval did not include zero. We estimated three models to test our hypotheses. Following Hamaker et al. (2023), we label (cross)-regressive effects of one variable predicting another as Beta ($\beta$), auto-regressive effects of a variable predicting itself as Phi ($\phi$), and residual variances as Psi ($\psi$). All models were pre-registered prior to conducting analyses (see https://osf.io/jz6bm).

#### Model 1: Effect of the Mindfulness Intervention on Rumination and NA Levels

**Fig. 1:**
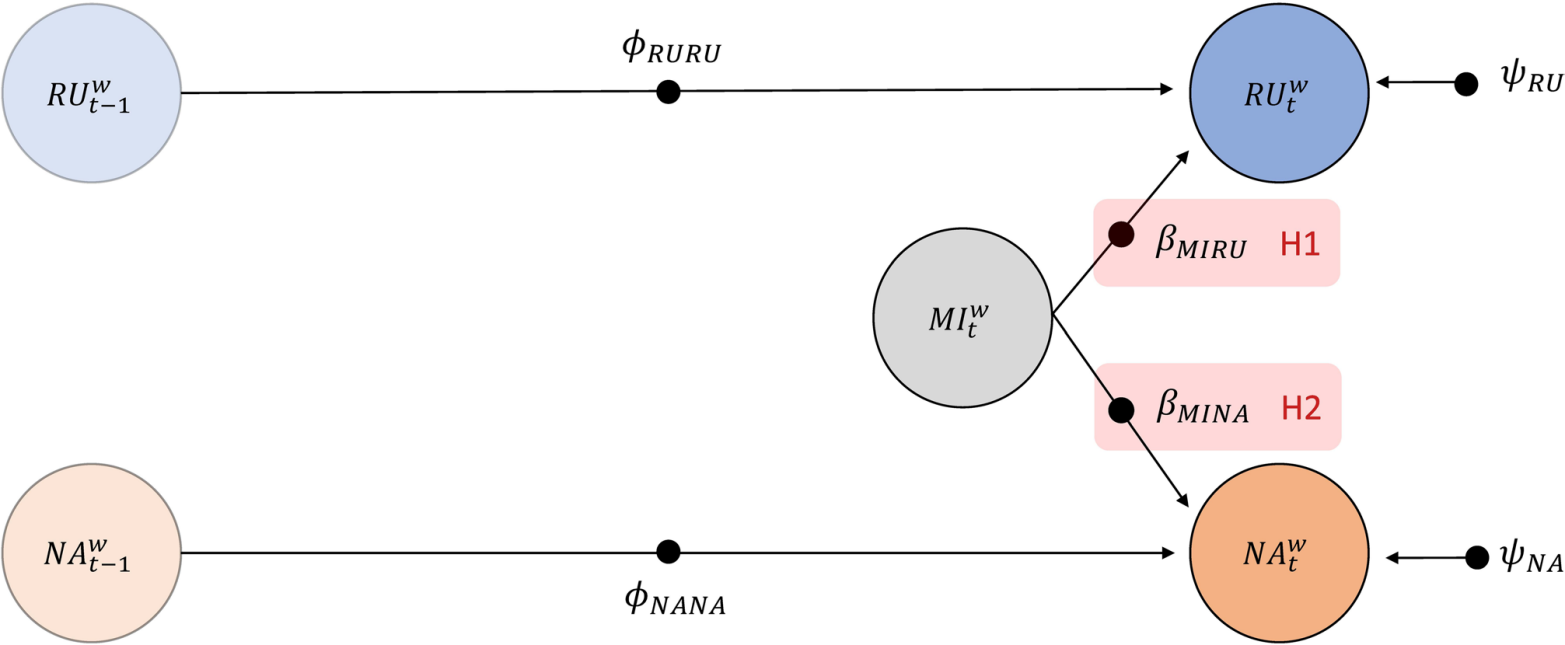
Within-person model investigating the effect of the mindfulness intervention on levels of rumination and NA. *Note.* The filled circles indicate that the parameters are random. RU = rumination, NA = negative affect, MI = mindfulness intervention, *w* = within-person component of DSEM model, ${\phi }_{RURU}$ = autoregressive effect of rumination, ${\phi }_{NANA}$= autoregressive effect of NA, ${\beta }_{MIRU}$ = effect of mindfulness intervention at *t* on rumination at *t*, ${\beta }_{MINA}$ = effect of mindfulness intervention at *t* on NA at *t*,${\psi }_{RU}$= residual variance of rumination,${\psi }_{NA}$= residual variance of NA. H1 = hypothesis 1, H2 = hypothesis 2

Model 1 tested the hypotheses that the mindfulness intervention would predict lower levels of rumination (H1) and NA (H2) relative to the active-control task. As shown in Fig. 1, rumination (${RU}_{t}^{w}$) and NA (${NA}_{t}^{w}$) at occasion *t* were regressed onto a binary mindfulness intervention variable (${MI}_{t}^{w}$; where 1 = mindfulness intervention and 0 = control task delivered at occasion *t*). The slopes ${\beta }_{MIRU}$ and ${\beta }_{MINA}$ represent causal effects of the mindfulness intervention on rumination and NA and therefore tested H1 and H2, respectively (see red shaded parameters in Fig. 1). We also controlled for the autoregressive effects of rumination (${\phi }_{RURU}$) and NA (${\phi }_{NANA}$) by predicting levels of rumination and NA at occasion *t* by their respective levels at the previous occasion *t*−1. Lastly, Model 1 included random residual variances for rumination (${\psi }_{RU}$) and NA (${\psi }_{NA}$) reflecting unexplained variance in each outcome after accounting for all predictors.

#### Model 2: Effect of the Mindfulness Intervention on the Cross-Regressive Effect of Rumination on NA

**Fig. 2:**
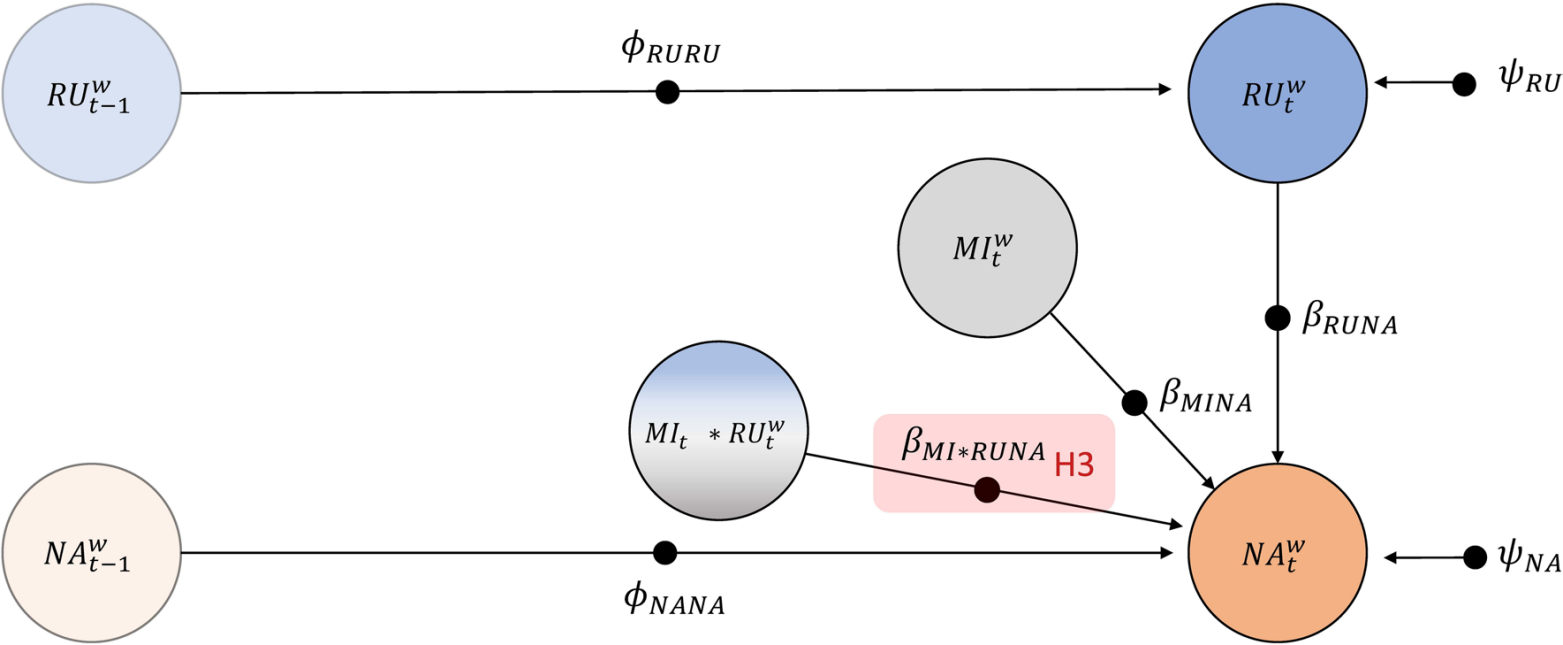
Within-Person model investigating the effect of the mindfulness intervention on the cross-regressive effect of rumination on NA. *Note.* The filled circles indicate that the parameters are random. RU = rumination, NA = negative affect, MI = mindfulness intervention, *w* = within-person component of DSEM model, ${\phi }_{RURU}$ = autoregressive effect of rumination, ${\phi }_{NANA}$= autoregressive effect of NA, ${\beta }_{RUNA}$ = cross-regressive effect of rumination at *t* on NA at *t*, ${\beta }_{MINA}$ = effect of mindfulness intervention at *t* on NA at *t*, ${\beta }_{MI*RUNA}$= effect of the mindfulness intervention at *t* on the cross-regressive effect of rumination at *t* on NA at *t*, ${\psi }_{RU}$= residual variance of rumination,${\psi }_{NA}$= residual variance of NA

Model 2 tested the hypothesis that the mindfulness intervention would predict a weaker cross-regressive effect of rumination on NA relative to the active control task (H3). As shown in Fig. 2, NA (${NA}_{t}^{w}$) at occasion *t* was regressed onto rumination at occasion t (${RU}_{t}^{w}$), the mindfulness intervention at occasion *t* (${MI}_{t}^{w}$), and their product (${MI}_{t}*{RU}_{t}^{w}$) representing the interaction between the mindfulness intervention and rumination. The slope ${\beta }_{MI*RUNA}$ represents the causal effect of the mindfulness intervention on the cross-regressive effect of rumination on NA and therefore tested H3 (see red shaded parameter in Fig. 2). Note that although NA is predicted by rumination measured at the same occasion (*t*), we consider rumination to be *conceptually lagged* because participants reported their rumination “over the last few minutes”. In contrast, participants reported their NA “right now” at each occasion *t*. Therefore, following Hamaker et al. (2023), we modelled how rumination predicted NA using a so-called *lag-0* cross-regressive effect, rather than a lag-1 effect. A recent simulation study suggests that this approach is a valid way of modelling causal effects among conceptually lagged and momentary variables (Luo & Hu, 2023). As in the previous model, we included autoregressive effects and residual variances for rumination and NA.

#### Model 3: Effect of the Mindfulness Intervention on the Cross-Regressive Effect of NA on Rumination and on the Autoregressive Effects of Rumination and NA

**Fig. 3:**
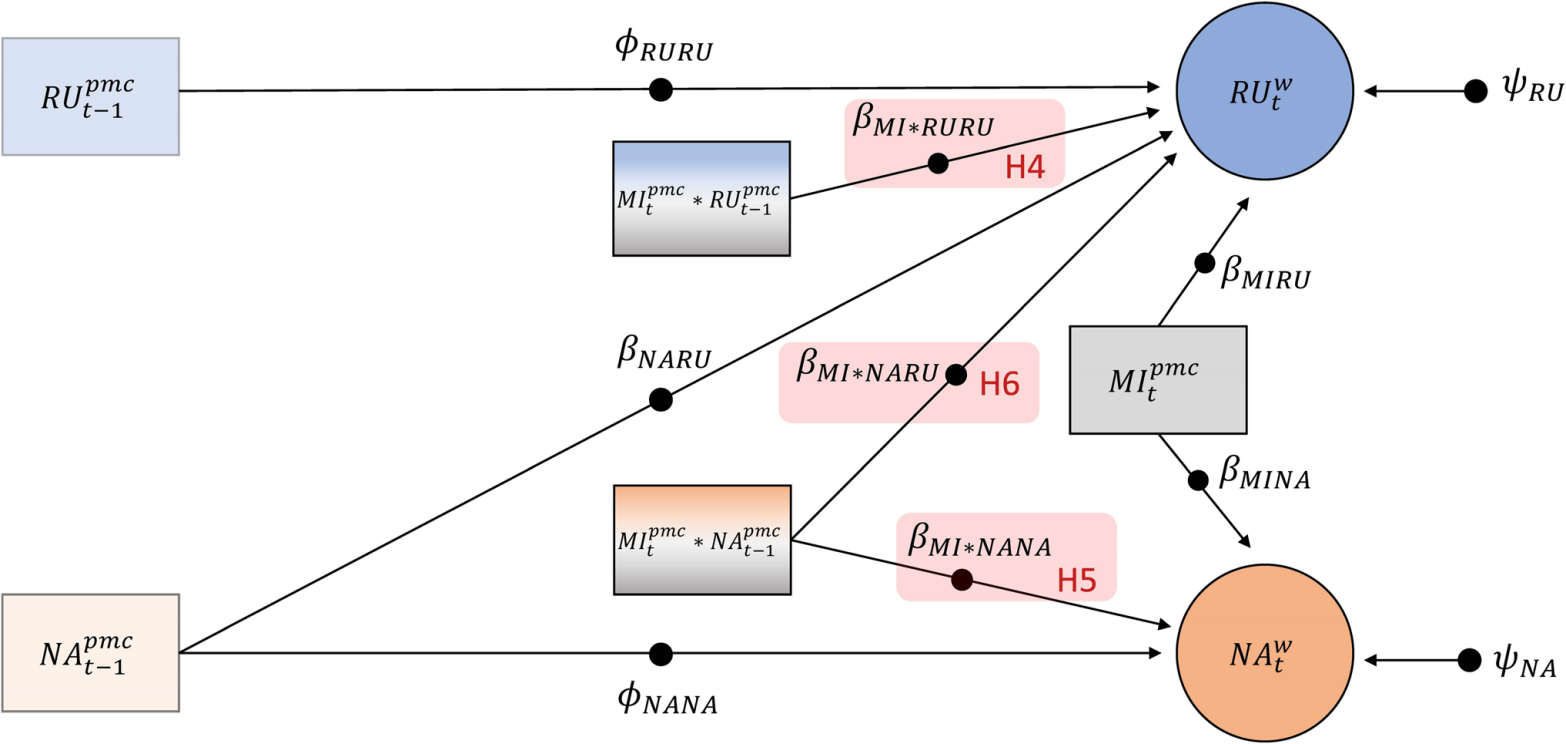
Within-Person model investigating the effect of the mindfulness intervention on the cross-regressive effect of NA on rumination and the autoregressive effects of rumination and NA. *Note.* The filled circles indicate that the parameters are random. RU = rumination, NA = negative affect, MI = mindfulness intervention, *w* = within-person component of DSEM model, *pmc* = observed person-mean centered, ${\phi }_{RURU}$ = autoregressive effect of rumination, ${\phi }_{NANA}$ = autoregressive effect of NA, ${\phi }_{NARU}$ = cross-regressive effect of NA at *t*−1 on rumination at *t*, ${\beta }_{MIRU}$ = effect of mindfulness intervention at *t* on rumination at *t*, ${\beta }_{MINA}$= effect of mindfulness intervention at *t* on NA at *t*, ${\beta }_{MI*NARU}$= effect of the mindfulness intervention on the cross-regressive effect of NA at *t*−1 on rumination at *t*, ${\beta }_{MI*RURU}$ = effect of the mindfulness intervention at *t* on the autoregressive effect of rumination, ${\beta }_{MI*NANA}$ = effect of the mindfulness intervention at *t* on the autoregressive effect of NA, ${\psi }_{RU}$= residual variance of rumination,${\psi }_{NA}$= residual variance of NA

Model 3 tested the hypothesis that, relative to the active-control task, the mindfulness intervention would weaken the cross-regressive effect of NA on rumination (H4), as well as the temporal persistence of rumination (H5) and NA (H6). As shown in Fig. 3, we regressed rumination at occasion *t* (${RU}_{t}^{pmc}$) onto NA at the *previous* occasion *t*−1 (${NA}_{t-1}^{pmc}$), the mindfulness intervention at occasion *t* (${MI}_{t}^{pmc}$) and their product (${MI}_{t}*{NA}_{t-1}^{pmc}$). The slope ${\beta }_{MI*NARU}$ represents the effect of the mindfulness intervention on the cross-regressive effect of NA on rumination and therefore tested H4 (see red shaded parameter in Fig. 3).

As in the previous models, we included autoregressive effects and residual variances for rumination and NA. Finally, to test hypotheses H5 and H6, we included interactions between the mindfulness intervention at occasion* t* and lagged rumination (${MI}_{t}^{pmc}*{RU}_{t-1}^{pmc}$) and NA (${MI}_{t}^{pmc}*{NA}_{t-1}^{pmc}$). Thus, the slopes ${\beta }_{MI*RURU}$ and ${\beta }_{MI*NANA}$ represent effects of the mindfulness intervention on the autoregressive effects of rumination and NA (testing H5 and H6, respectively; see red shaded parameters in Fig. 3). Because latent centering is currently not possible when modelling within level interactions that include lagged variables, we entered observed mean-centered predictors in Model 3, represented as rectangles in Fig. 3 (see Supplemental Material for more details).

## Results

For all analyses, we report unstandardized fixed effects (medians of the posterior distribution for each parameter) and their 95% credible intervals. For our main analyses we also report within-person standardized parameter estimates in Tables S2-S4 in the Supplemental Material.

### Preliminary Analyses

#### Descriptive Statistics

Participants answered an average of 79.2% (*SD* = 13.5, range = 33.3–98.8) of all delivered ESM surveys, yielding a total of 5,691 completed ESM surveys. On average, participants were randomized to complete the mindfulness intervention (vs. active-control task) on 49.8% (*SD* = 15, range = 17.6–87.2) of all completed ESM surveys. Participants spent the required three minutes listening to the audio tasks on 90.7% (*SD* = 9.67, range = 62.0–100) of completed surveys, on average. We checked this compliance with the audio tasks by inspecting the reaction time to the item during which participants were supposed to complete the audio task. Preliminary null models indicated that, on average, participants reported moderate levels of rumination and state mindfulness, but relatively low levels of NA across the ESM phase (see Table 2). All ESM measures showed substantial variation within and between persons, with ICCs between .48 and .67.

**Table 2:** Descriptive statistics for ESM measures

	*M*	*SD*_W_	*SD*_B_	*ICC*
Rumination	36.06 [32.55, 39.95]	18.59 [18.26, 18.93]	17.89 [15.21, 20.67]	.48 [.41, .56]
Negative Affect	17.04 [14.22, 20.32]	10.34 [10.18, 10.53]	14.69 [12.49, 16.94]	.67 [.60, .73]
State mindfulness	52.02 [48.58, 55.77]	13.35 [13.11, 13,60]	17.39 [14.97, 20.19]	.63 [.56, .70]

Descriptive statistics are based on null models estimated in Mplus. *M* mean, *SD*_W_ within-person standard deviation, *SD*_B_ between-person standard deviation, *ICC* intraclass correlation, which represents the ratio of between-person to total variation for each measure. Values in square brackets are 95% Bayesian credibility intervals

#### Manipulation Check

We tested whether the mindfulness intervention induced higher levels of state mindfulness by regressing state mindfulness at each occasion *t* onto the mindfulness intervention variable at each occasion *t* (see Supplemental Material for detailed model and results). This analysis indicated that the mindfulness intervention was successful in inducing increases in state mindfulness: On average, participants reported significantly higher mindfulness after completing the mindfulness intervention versus the active-control task (*b* = 3.38, 95% *CI* = [2.39, 4.36]). Neubauer et al. (2024) provide additional findings on the effects of the intervention on state mindfulness. For instance, they report that the extent to which participants' state mindfulness increased via each intervention was unrelated to how often they received the intervention.

### Main Analyses

#### Model 1

Results of Model 1 (see Table 3) showed that the average effect of the mindfulness intervention on rumination was significantly negative (see ${\beta }_{MIRU}$ slope). Thus, consistent with H1, participants reported levels of rumination that were approximately 2.7 points lower (on a 0–100 scale) after completing the mindfulness intervention as compared with the active-control task. Similarly, consistent with H2, the effect of the mindfulness intervention on NA was significantly negative (see ${\beta }_{MINA}$ slope), implying that participants reported levels of NA that were approximately 1.2 points lower after completing the mindfulness intervention as compared with the active-control task.

**Table 3:** Unstandardized fixed effects (median) from model 1

	*Estimate*	*95% CI*	
Parameters		*Lower*	*Upper*
RU intercept	36.016	32.308	39.777
NA intercept	15.875	13.481	18.201
*Autoregressive effects*			
${\phi }_{RURU}$	0.204	0.152	0.256
${\phi }_{NANA}$	0.375	0.317	0.432
*Effects of mindfulness intervention*			
${\beta }_{MIRU}$	−2.713	−3.986	−1.455
${\beta }_{MINA}$	−1.203	−1.725	−0.696
*Log residual variances*			
${\psi }_{RU}$	5.407	5.243	5.568
${\psi }_{NA}$	3.886	3.653	4.112

*CI* credible interval, *RU* rumination, *NA* negative affect, *MI* mindfulness intervention, ${\phi }_{RURU}$ autoregressive effect of rumination, ${\phi }_{NANA}$ autoregressive effect of NA, ${\beta }_{MIRU}$ effect of mindfulness intervention at *t* on rumination at *t*; ${\beta }_{MINA}$ effect of mindfulness intervention at *t* on NA at *t*, ${\psi }_{RU}$ log transformed residual variance of rumination,${\psi }_{NA}$ log transformed residual variance of NA

#### Model 2

Results of Model 2 (see Table 4) showed that the mindfulness intervention did not significantly moderate the cross-regressive effect of rumination on NA (see ${\beta }_{MI*RUNA}$ slope). Thus, contrary to H3, the mindfulness intervention did not weaken the effect of rumination on NA. In fact, contrary to our hypothesis, the standardized estimate of $\beta$_MI*RUNA_ was significant and positive (*Est*. = 0.082, 95% *CI* = [0.026, 0.143]), suggesting that the mindfulness intervention may have strengthened the effect of rumination on NA (see Table S3 in the Supplemental Material for full standardized model results). As we discuss further below, we urge caution in interpreting this effect given the discrepancy between its raw and standardized estimates.

**Table 4:** Unstandardized fixed effects (median) from model 2

	*Estimate*	*95% CI*	
Parameters		*Lower*	*Upper*
RU intercept	35.962	32.318	39.545
NA intercept	15.971	13.526	18.327
*Autoregressive effects*			
${\phi }_{RURU}$	0.206	0.151	0.260
${\phi }_{NANA}$	0.317	0.259	0.374
*Cross-regressive effects*			
${\beta }_{RUNA}$	0.159	0.117	0.201
*(Interaction) Effects of mindfulness intervention*			
${\beta }_{MINA}$	−1.200	−1.834	−.588
${\beta }_{MI*RUNA}$	0.027	−0.011	0.065
*Log residual variances*			
${\psi }_{RU}$	5.559	5.405	5.710
${\psi }_{NA}$	3.738	3.511	3.970

*CI* credible interval, *RU* rumination, *NA* negative affect, *MI* mindfulness intervention, ${\phi }_{RURU}$ autoregressive effect of rumination, ${\phi }_{NANA}$ autoregressive effect of NA, ${\beta }_{RUNA}$ cross-regressive effect of rumination at *t* on NA at *t*, ${\beta }_{MINA}$ effect of mindfulness intervention at *t* on NA at *t*, ${\beta }_{MI*RUNA}$ effect of the mindfulness intervention at *t* on the cross-regressive effect of rumination at *t* on NA at *t*, ${\psi }_{RU}$ log transformed residual variance of rumination, ${\psi }_{NA}$ log transformed residual variance of NA

#### Model 3

Results of Model 3 (see Table 5) showed that the mindfulness intervention did not significantly moderate the cross-regressive effect of NA on rumination (see ${\beta }_{MI*NARU}$ slope). Thus, contrary to H4, previous NA was not less strongly associated with rumination when the mindfulness intervention was completed as compared to the active-control task. Similarly, the mindfulness intervention did not significantly moderate the autoregressive effects of rumination or NA (see ${\beta }_{MI*RURU}$ and ${\beta }_{MI*NANA}$ slopes, respectively). Thus, contrary to H5 and H6, rumination and NA were not less persistent when the mindfulness intervention was completed as compared to the active-control task.

**Table 5:** Unstandardized fixed effects (median) of model 3

	*Estimate*	*95% CI*	
Parameters		*Lower*	*Upper*
RU intercept	36.009	31.704	40.503
NA intercept	17.003	13.462	20.557
*Autoregressive effects*			
${\phi }_{RURU}$	0.156	0.100	0.210
${\phi }_{NANA}$	0.338	0.276	0.397
*Cross-regressive effects*			
${\phi }_{NARU}$	0.159	0.117	0.201
*(Interaction) Effects of mindfulness intervention*			
${\beta }_{MIRU}$	−4.181	−6.713	−1.713
${\beta }_{MINA}$	−1.518	−2.491	−0.629
${\beta }_{MI*NARU}$	0.041	−0.142	0.230
${\beta }_{MI*RURU}$	0.014	−0.085	0.114
${\beta }_{MI*NANA}$	0.010	−0.081	0.103
*Log residual variances*			
${\psi }_{RU}$	5.451	5.255	5.638
${\psi }_{NA}$	3.942	3.660	4.205

*CI* credible interval, *RU* rumination, *NA* negative affect, *MI* mindfulness intervention, ${\phi }_{RURU}$ autoregressive effect of rumination, ${\phi }_{NANA}$ autoregressive effect of NA, ${\phi }_{NARU}$ cross-regressive effect of NA at *t*−1 on rumination at *t*, ${\beta }_{MIRU}$ effect of mindfulness intervention at *t* on rumination at *t*, ${\beta }_{MINA}$ effect of mindfulness intervention at *t* on NA at *t*, ${\beta }_{MI*NARU}$ effect of the mindfulness intervention at *t* on the cross-regressive effect of NA at *t*−1on rumination at *t*, ${\beta }_{MI*RURU}$ effect of the mindfulness intervention at *t* on the autoregressive effect of rumination,${\beta }_{MI*NANA}$ effect of the mindfulness intervention at *t* on the autoregressive effect of NA, ${\psi }_{RU}$ log transformed residual variance of rumination, ${\psi }_{NA}$ log transformed residual variance of NA

### Supplemental Analyses

We ran a series of additional analyses designed to test the robustness of our main findings, by (1) varying our approach of dealing with intervention non-compliance, (2) varying our approach of dealing with unequal time intervals, and (3) including random residual covariances among outcome variables. Models with these alternative specifications resulted in very similar findings supporting the same conclusions as our main analyses. Further details and model results from these additional analyses are provided in the Supplemental Material (see Table S5-S7).

## Discussion

Mindfulness interventions are often promoted as helpful in alleviating psychological distress and interrupting mutually and self-reinforcing dynamic associations among negative thoughts and feelings. However, previous studies were not able to test the within-person causal effect of engaging in brief mindfulness exercises in daily life on rumination, NA, and their dynamics. We investigated this possibility using a micro-randomized trial, in which participants received a mindfulness intervention and an active-control task at random moments in their daily lives and then reported their experiences of rumination and NA.

Results of our first model revealed that the mindfulness intervention predicted lower levels of rumination and NA, consistent with our hypotheses and with results of previous observational ESM studies (Blanke et al., 2018, 2020; Brown & Ryan, 2003). Due to random assignment of the mindfulness intervention (vs. active-control task) at each occasion, our findings extend upon previous research by providing the first evidence of a within-person causal effect of mindfulness on rumination and NA in daily life. Previous studies have combined experimental designs with ESM to study the causal impact of mindfulness on daily life psychological experience (e.g., Bolzenkötter et al., 2024; Rowland et al., 2020). However, these studies randomized individuals, not measurement occasions, to a mindfulness (vs. control) intervention. Results of previous ESM studies therefore represent between-person effects of mindfulness, which cannot necessarily be generalized to the within-person level (Molenaar, 2004; Neubauer et al., 2024).

However, contrary to our hypotheses, we found no consistent evidence that the mindfulness intervention influenced the temporal dynamics of rumination and NA. Specifically, results of our second model were inconsistent with our prediction that mindfulness would weaken the cross-regressive effect of rumination on NA. This also diverges from the results of Blanke et al.’s (2020) observational ESM study. In fact, standardized estimates from our second model suggest that the mindfulness intervention may have strengthened the cross-regressive effect of rumination on NA. To understand this unexpected finding, consider that the standardized results are based on within-person standardized variables, which explicitly remove between-person differences in means and variances. This suggests that between-person differences in variability of rumination and/or NA may have reduced the reliability of the unstandardized interaction effect, which was also positive but had a 95% CI that crossed zero (compare Rowland et al., 2020 for a similar divergent finding between standardized and non-standardized results). In sum, although we urge caution in interpreting the positive standardized interaction effect, we can be relatively confident that our results do not support the prediction that inducing mindfulness *weakens* the effect of rumination on NA.

Finally, results of our third model were inconsistent with our hypotheses that the mindfulness intervention would weaken the effect of NA on subsequent rumination, as well as the inertia of rumination and NA. Note, however, that unlike the first two models, here we estimated lagged effects across successive ESM surveys. Thus, our findings suggest that the mindfulness intervention did not impact how much previous experiences of NA predicted themselves or rumination across a time interval of roughly 90 min. This may be explained by the relatively long timespan between consecutive measurement occasions surveys combined with the brevity of the mindfulness intervention. We note, however, that our findings are consistent with Rowland et al. (2020), who found no within-person moderating effect of momentary mindfulness on NA inertia.

### Limitations and Future Directions

Our study has limitations that may be addressed by future research. First, participants completed both the mindfulness intervention and control tasks in our study. This may have induced a demand effect whereby participants expected the mindfulness intervention to be more effective. This is less of an issue in between-person experimental designs, where participants are randomized to either a treatment or control condition. Future studies using within-person designs could mitigate this concern by attempting to equalize participants’ expectations about the effectiveness of the mindfulness and control interventions.

Second, we compared a brief mindfulness intervention with a control task comprising neutral background noise. Thus, our findings might not generalize to other forms of mindfulness practice or to other control tasks. For example, Bolzenkötter et al. (2024) found that a detached mindfulness intervention and a guided-imagery control task – matched with the mindfulness task on all features except mindfulness-specific content –had comparable effects on rumination and NA in daily life. This challenges the specific role of mindfulness as the mechanism of action, leaving open the possibility that mindfulness-unspecific factors (e.g., expected benefits, distraction) may have accounted for the observed reductions in rumination and NA. In contrast, in the current study, the mindfulness intervention resulted in significantly lower rumination and NA than the control task. Yet, because our two tasks differed on several mindfulness-unspecific dimensions (e.g., no verbal instruction in the control task), we cannot be sure which ingredient(s) of the mindfulness intervention were responsible for the salutary effects we observed.

Thirdly, future studies may also explore the effects of a longer mindfulness intervention or use a denser ESM design with shorter time intervals between measurement occasions. Such designs could reveal more fleeting effects of mindfulness on the dynamics of psychological experience, which did not emerge in the current study.

Lastly, other characteristics of our study may limit the generalizability of our findings. Data were collected during the COVID-19 pandemic and our sample can be considered relatively young and highly educated. Future research is needed to determine whether our findings generalize to non-pandemic times and among other, more diverse samples. Additionally, completing the mindfulness intervention and answering ESM surveys might have been more feasible for participants with greater time flexibility (e.g., working or studying from home). Therefore, our findings may not generalize to individuals in less flexible employment or whose life circumstances prevent them from engaging in a brief (mindfulness) exercise several times each day. Finally, our study investigated the immediate effect of one specific brief audio-guided mindfulness intervention. The effects of this intervention may not generalize to more comprehensive mindfulness-based treatment programs, which typically include a variety of different interventions/exercises administered over several weeks. Research on the efficacy of such comprehensive mindfulness programs typically focuses on assessing change in outcomes over longer timescales (i.e., pre- to post-treatment) rather than on the short-term effects of completing a single intervention or exercise.

## Conclusion

This is the first study to experimentally manipulate participants’ state mindfulness in daily life and investigate the within-person impact on levels and temporal dynamics of rumination and NA. Our results suggest that inducing state mindfulness via a brief exercise leads to less rumination and NA in the immediate short-term. Increasing peoples’ mindfulness in daily life may therefore have short-term benefits for mental well-being. Our results further suggest that moments of higher state mindfulness do not reliably lead to weaker mutually and self-reinforcing dynamics among rumination and NA. Thus, brief mindfulness practices appear insufficient to disrupt the dynamical relations between rumination and NA.

## Supplementary Information

Below is the link to the electronic supplementary material.

Supplementary file1 (DOCX 757 KB)

## Additional Information

### Data Availability

All data, analysis code, and research materials are publicly available at https://osf.io/y3gnt/ (see folder “Momentary Mindfulness and Rumination”).

### Code Availability

Code is available at https://osf.io/y3gnt/ (see folder “Momentary Mindfulness and Rumination”).
