# Supplementary material for: Impact of a Momentary Mindfulness Intervention on Rumination, Negative Affect, and their Dynamics in Daily Life
Source: Affect Sci. 2025 Jan 24;6(2):259–71. doi: 10.1007/s42761-024-00291-9 (PMC12209136; doi:10.1007/s42761-024-00291-9)
Supplement: Supplementary file 1 — Supplementary file1 (DOCX 757 KB) [file 42761_2024_291_MOESM1_ESM.docx]

Supplemental Material

Contents

[Participant Inclusion Criteria 2](#_Toc161471765)

[Flow of Participants 2](#_Toc161471766)

[Cross-Classified Variance Decomposition Models 3](#_Toc161471767)

[Additional Information for Main Analyses Testing Hypotheses 4](#_Toc161471768)

[Explanation of Variables and Model Diagrams 4](#_Toc161471769)

[Model 1 5](#_Toc161471770)

[Model 2 7](#_Toc161471771)

[Model 3 8](#_Toc161471772)

[Standardized Model Results 10](#_Toc161471773)

[Model 1 10](#_Toc161471774)

[Model 2 11](#_Toc161471775)

[Model 3 12](#_Toc161471776)

[Manipulation Check 13](#_Toc161471777)

[Supplemental Analyses 14](#_Toc161471778)

[Literature 20](#_Toc161471779)

# Participant Inclusion Criteria

Our inclusion criteria diverge from what we stated in our original study pre-registration as follows: the pre-registration does not mention vision, hearing, or mental health exclusions. Further, the pre-registration mentions prior mindfulness training experience as an exclusion, however, this was not applied. We instead measured previous meditation experience and practice frequency (see Table 1).

# Flow of Participants

Figure S1

*Flowchart of Participants*


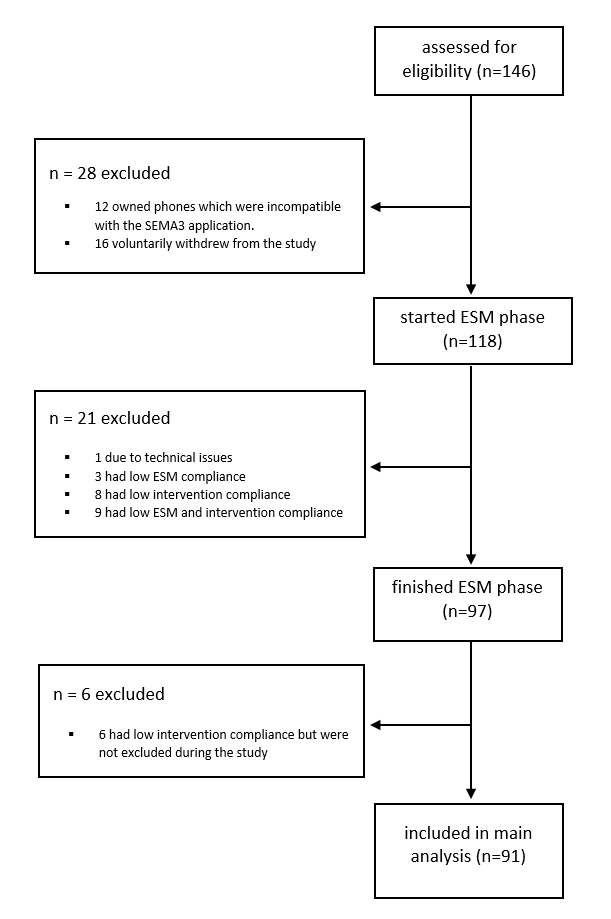


# Cross-Classified Variance Decomposition Models

| Table S1  *Cross-Classified Variance Decomposition Models* | | | | | | | | | |
| --- | --- | --- | --- | --- | --- | --- | --- | --- | --- |
|  | Level 1  (within person/occasion) | |  | Between Persons | |  | Between Occasions | |  |
| Variable | Variance | VPC |  | Variance | VPC |  | Variance | VPC |  |
| Rumination | 345.443 | .519 |  | 319.940 | .480 |  | 0.501 | .001 |  |
| NA | 102.308 | .317 |  | 215.595 | .667 |  | 4.913 | .015 |  |
| Mindfulness | 178.149 | .370 |  | 302.015 | .629 |  | 0.321 | .001 |  |
| *Note*. VPC = variance partition coefficient, NA = negative affect. | | | | | | | | | |

Results of empty or “null” cross-classified models used to estimate the proportion of variance in each of our ESM measures are shown in Table S2 (above). Results of these models indicated that less than 1% of the total variance in rumination and mindfulness, and less than 2% of the variance in NA was between occasions. In contrast, approximately 33-67% of the total variance in each outcome was at the between-person and within-person/occasion levels. In light of the negligible amount of variance at the between-occasion level, we opted to test our hypotheses using two-level models.

# Additional Information for Main Analyses Testing Hypotheses

## Explanation of Variables and Model Diagrams

The Figures S2-S10 (see below) include the latent decomposition, the within-person model, and the between-person model of our dynamics structural equation modeling (DSEM) analyses conducted as main analyses to test our hypotheses.

In the model diagrams, we label parameters as follows:

- $\phi$ = auto-regressive slope
- $\beta$ = cross-regressive slope
- $\psi$ = innovation variance

We include the following variables in our models, with subscripts $i$ and $t$ represent people and occasions, respectively:

- ${NA}_{ti}$ = person $i$’s negative affect score at occasion $t$
- ${RU}_{ti}$ = person $i$’s rumination score at occasion $t$
- ${MI}_{ti}$ = a binary predictor, coded as 1 when the mindfulness intervention was delivered and 0 when the control task was delivered for each person $i$ at each occasion *t*.
- ${MI}_{ti}*{RU}_{ti}$ = an interaction term calculated as the product of ${MI}_{ti}$ and ${RU}_{ti}$

These observed variables are decomposed into orthogonal within and between person components using latent centering, as reflected by the superscripts $w$ and $b$, e.g., the latent within and between components of ${NA}_{ti}$ are ${NA}_{t}^{w}$ and ${NA}_{i}^{b}$, respectively. However, latent centering is not possible when including interactions between an observed and a latent lagged variable, as in Model 3 (see below). Therefore, we used observed-mean centering in Model 3. To do so, we (1) person-mean centered RU, NA, and the mindfulness intervention using their observed person-specific means, (2) created the lag 1 variables for RU and NA using the person-mean centered variables, and (3) created the interaction term between these lagged variables and the person-mean centered mindfulness intervention variable. Consequently, in Model 3, we included the following observed person-mean centered variables (indicated by the superscript $pmc$):

- ${NA}_{t-1}^{pmc}$ = person-mean centered negative affect at occasion $t-1$
- ${RU}_{t-1}^{pmc}$ = person-mean centered rumination at occasion $t-1$
- ${MI}_{t}^{pmc}$ = person-mean centered mindfulness intervention (vs. control task) delivered at occasion *t*
- ${MI}_{t}^{pmc}*{NA}_{t-1}^{pmc}$ = an interaction term calculated as the product of ${MI}_{t}^{pmc}$ and ${NA}_{t-1}^{pmc}$
- ${MI}_{t}^{pmc}*{RU}_{t-1}^{pmc}$ = an interaction term calculated as the product of ${MI}_{t}^{pmc}$ and ${RU}_{t-1}^{pmc}$

### Model 1

Figure S2

*Latent Decomposition of Model 1*


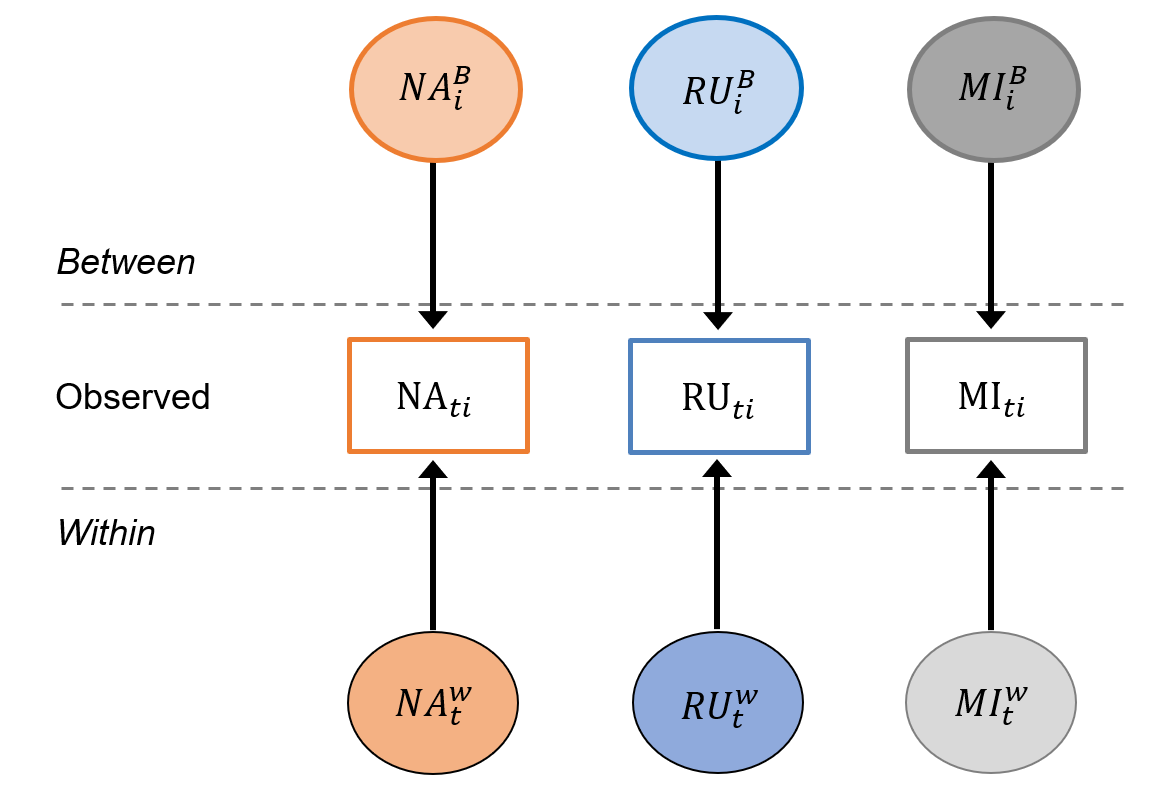


*Note*. RU = rumination, NA = negative affect, MI = mindfulness intervention, *W* = within-person component of DSEM model, *B* = between-person component of DSEM model, *i* = individual *i*, *t* = occasion *t*

Figure S3

*Within-Person Model of Model 1*


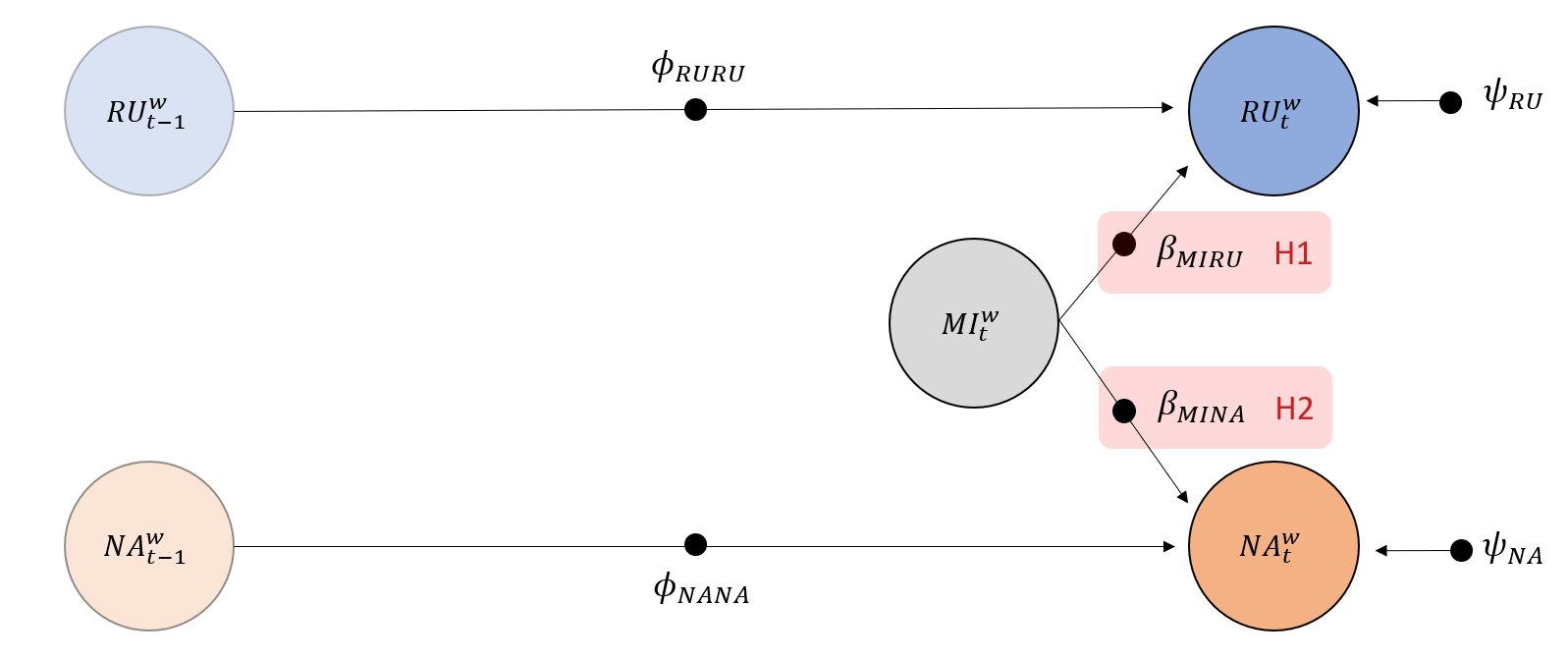


*Note*. RU = rumination, NA = negative affect, MI = mindfulness intervention, *w* = within-person component of DSEM model, $\phi_{RURU}$ = autoregressive effect of rumination, $\phi_{NANA}$= autoregressive effect of negative affect, $\beta_{MIRU}$ = effect of mindfulness intervention at *t* on rumination at *t*, $\beta_{MINA}$ = effect of mindfulness intervention at *t* on negative affect at *t*, $\psi_{RU}$= residual variance of rumination,$\psi_{NA}$= residual variance of negative affect. The filled circles indicate that the parameters are random.

Figure S4

*Between-Person Model of Model 1*


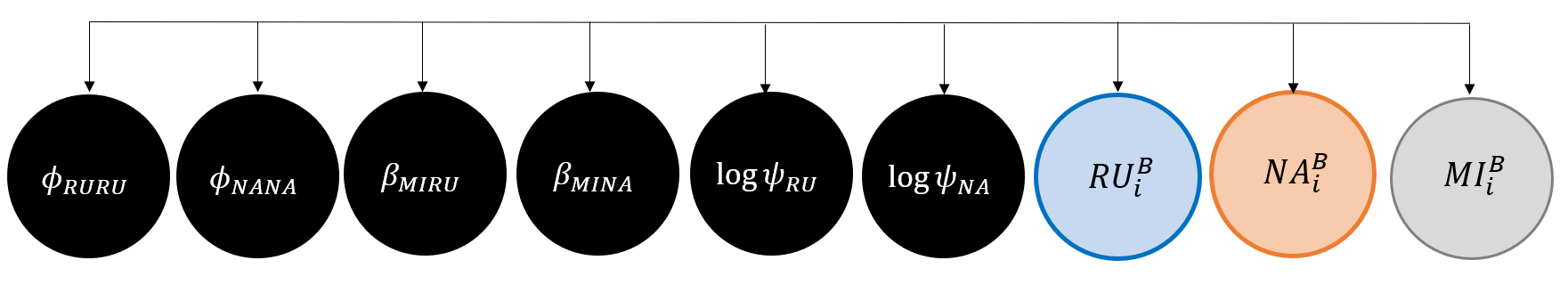


*Note*. RU = rumination, NA = negative affect, MI = mindfulness intervention, *B* = between-person component of DSEM model, *i* = individual *i*, $\phi_{RURU}$ = autoregressive effect of rumination, $\phi_{NANA}$= autoregressive effect of negative affect, $\beta_{RUNA}$ = cross-regressive effect of rumination at *t* on negative affect at *t*, $\beta_{MINA}$ = effect of mindfulness intervention at *t* on negative affect at *t*, $\beta_{MI*RUNA}$= effect of the mindfulness intervention at *t* on the cross-regressive effect of rumination at *t* on negative affect at *t*, ${log \psi}_{RU}$= log-transformed residual variance of rumination,${log\psi}_{NA}$= log-transformed residual variance of negative affect.

### Model 2

Figure S5

*Latent Decomposition of Model 2*


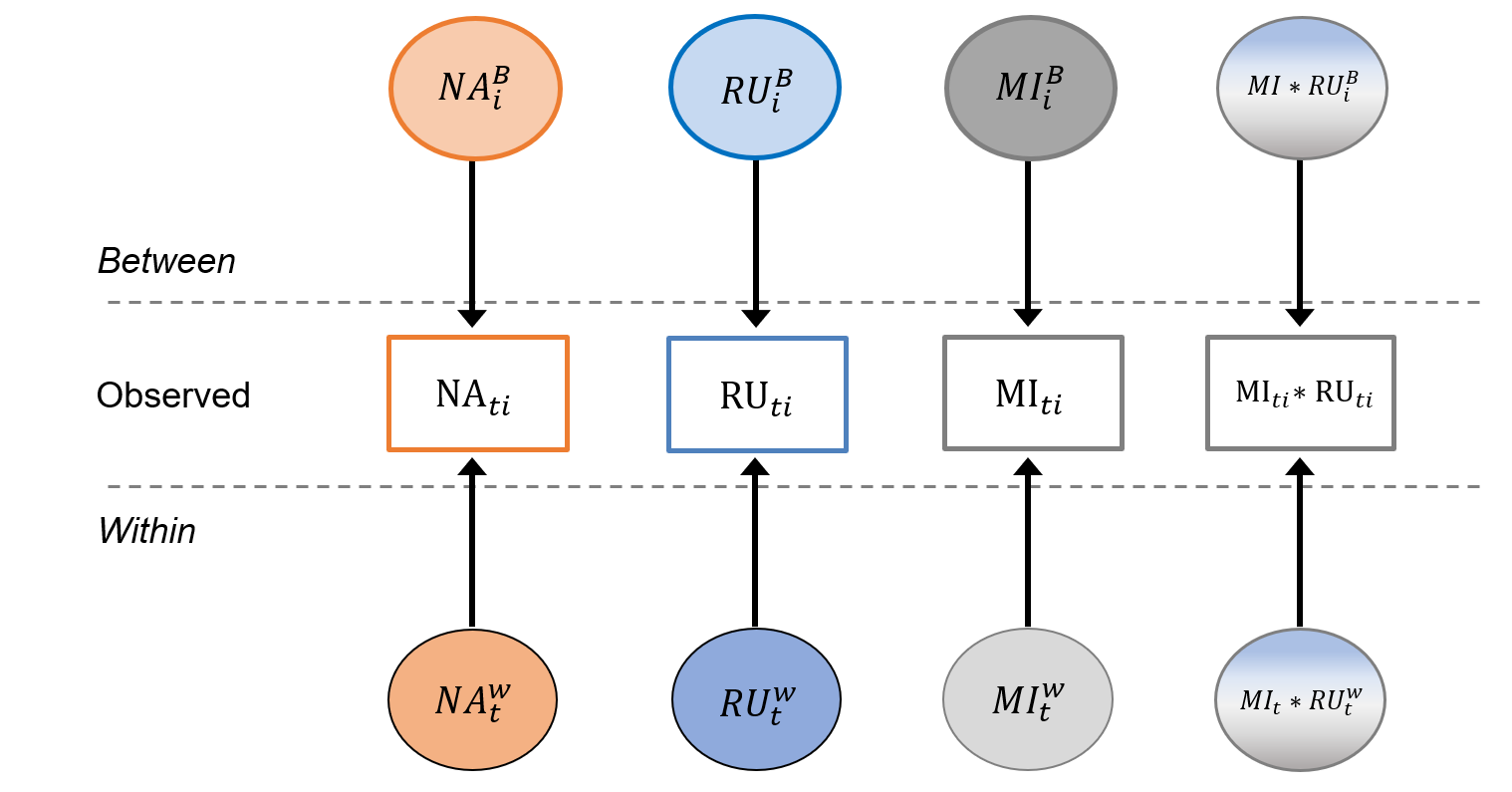


*Note*. RU = rumination, NA = negative affect, MI = mindfulness intervention, *W* = within-person component of DSEM model, *B* = between-person component of DSEM model, *i* = individual, *t* = occasion *t.*

Figure S6

*Within-Person Model of Model 2*


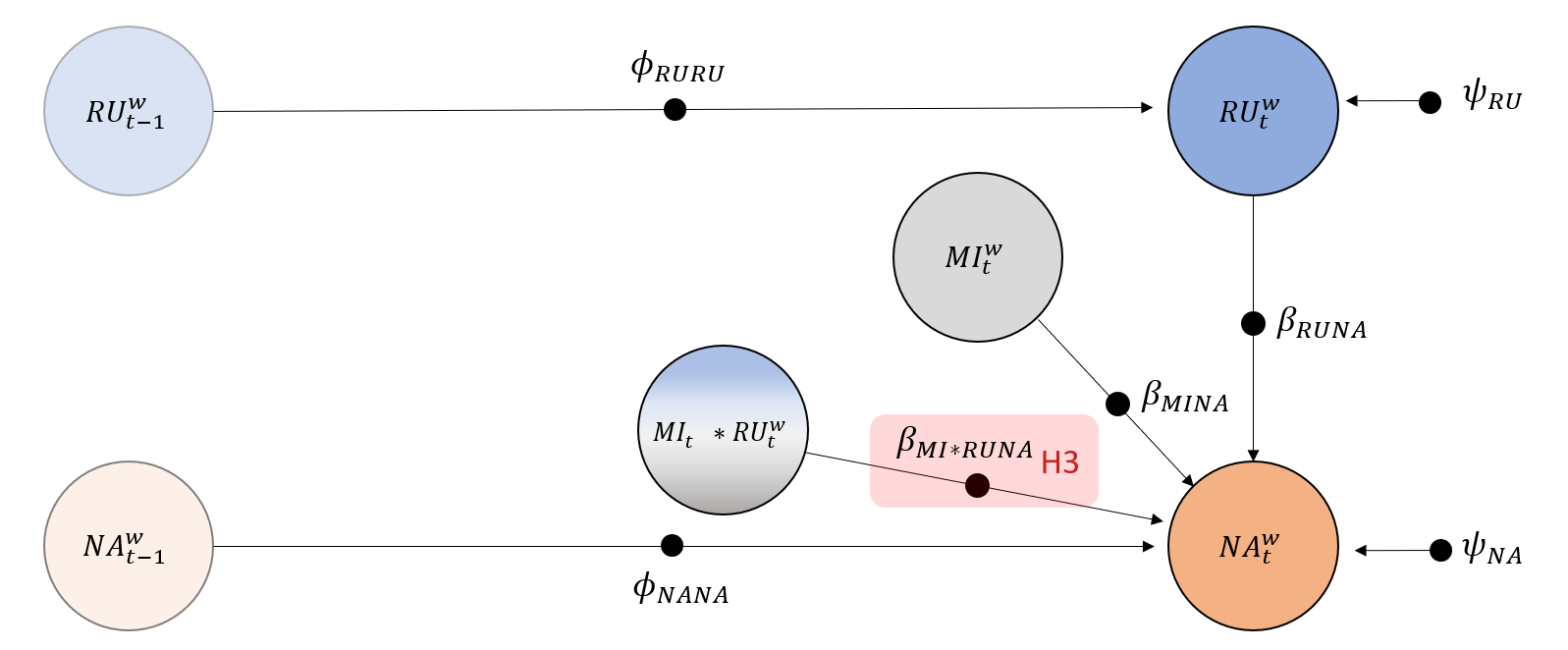


*Note*. RU = rumination, NA = negative affect, MI = mindfulness intervention, *w* = within-person component of DSEM model, $\phi_{RURU}$ = autoregressive effect of rumination, $\phi_{NANA}$= autoregressive effect of negative affect, $\beta_{RUNA}$ = cross-regressive effect of rumination at *t* on negative affect at *t*, $\beta_{MINA}$ = effect of mindfulness intervention at *t* on negative affect at *t*, $\beta_{MI*RUNA}$= effect of the mindfulness intervention at *t* on the cross-regressive effect of rumination at *t* on negative affect at *t*, $\psi_{RU}$= residual variance of rumination,$\psi_{NA}$= residual variance of negative affect. The filled circles indicate that the parameters are random.

Figure S7

*Between-Person Model of Model 2*


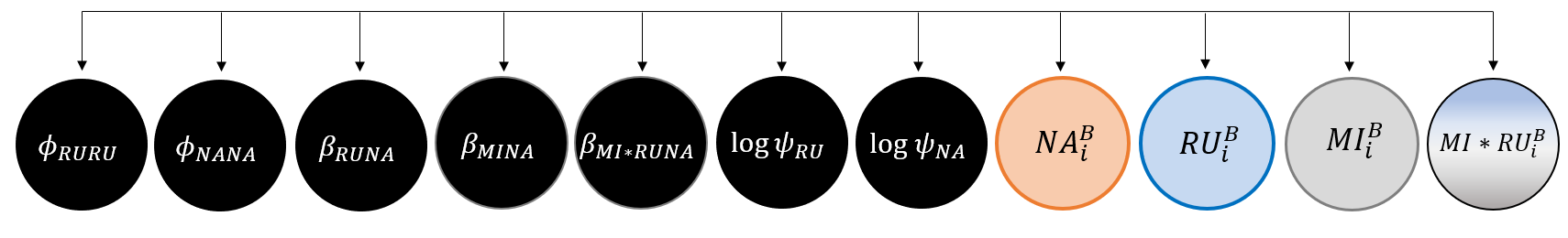


*Note*. RU = rumination, NA = negative affect, MI = mindfulness intervention, *B* = between-person component of DSEM model, *i* = individual *i*, $\phi_{RURU}$ = autoregressive effect of rumination, $\phi_{NANA}$= autoregressive effect of negative affect, $\beta_{RUNA}$ = cross-regressive effect of rumination at *t* on negative affect at *t*, $\beta_{MINA}$ = effect of mindfulness intervention at *t* on negative affect at *t*, $\beta_{MI*RUNA}$= effect of the mindfulness intervention at *t* on the cross-regressive effect of rumination at *t* on negative affect at *t*, ${log\psi}_{RU}$= log-transformed residual variance of rumination,$\log\psi_{NA}$= log-transformed residual variance of negative affect.

### Model 3

Figure S8

*Latent Decomposition of Model 3*


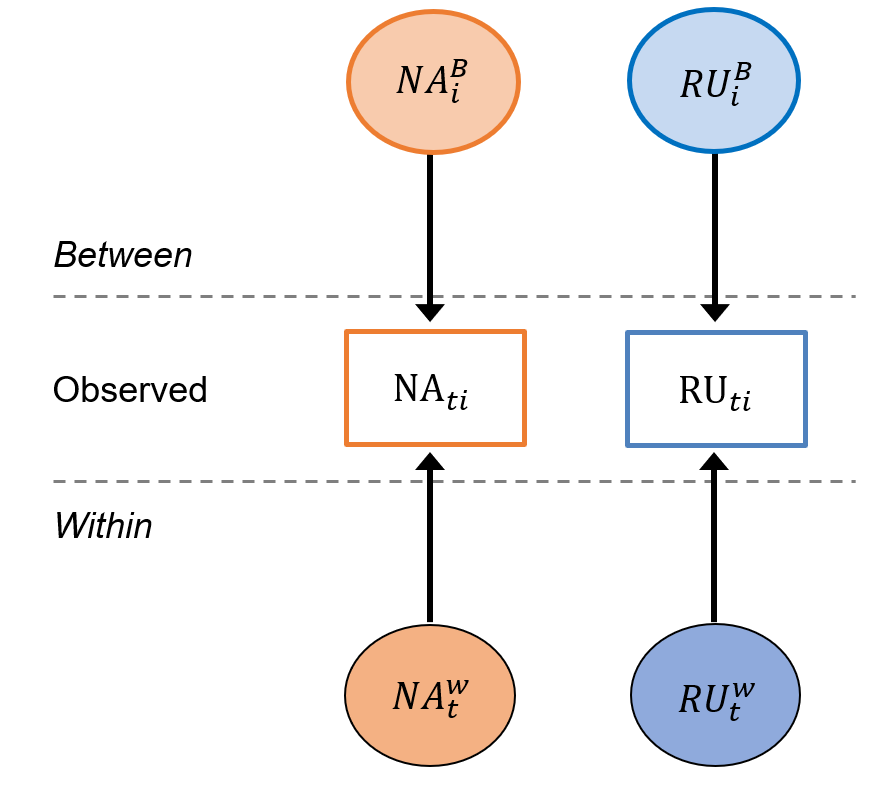


*Note*. RU = rumination, NA = negative affect, *W* = within-person component of DSEM model, *B* = between-person component of DSEM model, *i* = individual *i*, *t* = occasion *t.*

Figure S9

*Within-Person Model of Model 3*


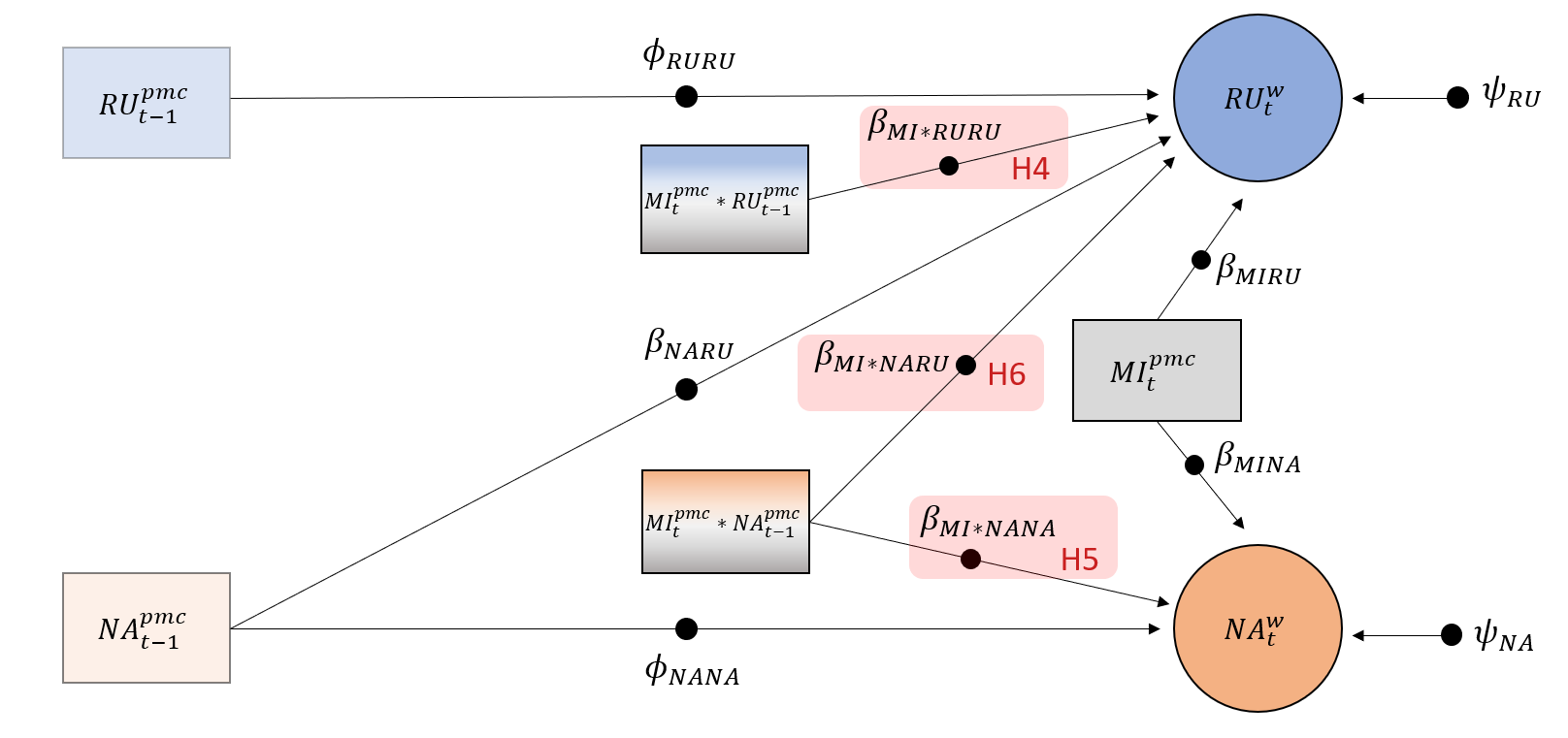


*Note*. RU = rumination, NA = negative affect, MI = mindfulness intervention, *w* = within-person component of DSEM model, *pmc* = observed person-mean centered, $\phi_{RURU}$ = autoregressive effect of rumination, $\phi_{NANA}$ = autoregressive effect of negative affect, $\phi_{NARU}$ = cross-regressive effect of negative affect at *t*-1 on rumination at *t*, $\beta_{MIRU}$ = effect of mindfulness intervention at *t* on rumination at *t*, $\beta_{MINA}$= effect of mindfulness intervention at *t* on negative affect at *t*, $\beta_{MI*NARU}$= effect of the mindfulness intervention on the cross-regressive effect of negative affect at *t*-1 on rumination at *t*, $\beta_{MI*RURU}$ = effect of the mindfulness intervention at *t* on the autoregressive effect of rumination, $\beta_{MI*NANA}$ = effect of the mindfulness intervention at *t* on the autoregressive effect of negative affect, $\psi_{RU}$= residual variance of rumination,$\psi_{NA}$= residual variance of negative affect. The filled circles indicate that the parameters are random.

Figure S10

*Between-Person Model of Model 3*


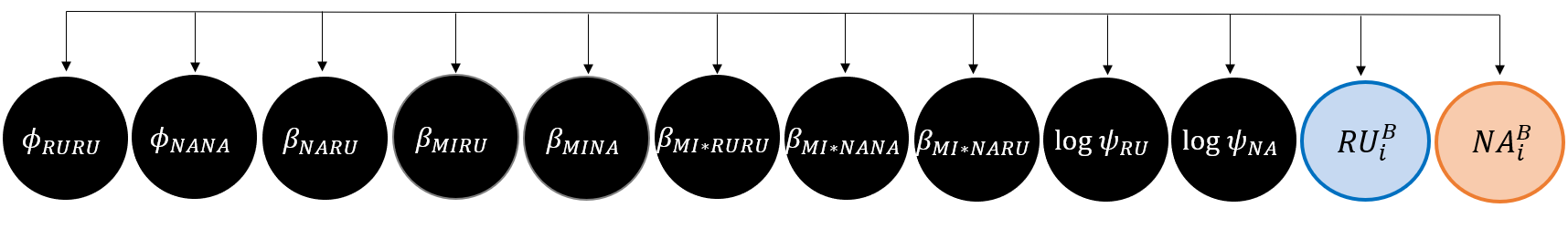


*Note*. RU = rumination, NA = negative affect, *B* = between-person component of DSEM model, *i* = individual *i*, $\phi_{RURU}$ = autoregressive effect of rumination, $\phi_{NANA}$= autoregressive effect of negative affect, $\phi_{NARU}$ = cross-regressive effect of negative affect at *t*-1 on rumination at *t*, $\beta_{MIRU}$ = effect of mindfulness intervention at *t* on rumination at *t*, $\beta_{MINA}$= effect of mindfulness intervention at *t* on negative affect at *t*, $\beta_{MI*RURU}$ = effect of the mindfulness intervention at *t* on the autoregressive effect of rumination, $\beta_{MI*NANA}$ = effect of the mindfulness intervention at *t* on the autoregressive effect of negative affect, $\beta_{MI*NARU}$= effect of the mindfulness intervention on the cross-regressive effect of negative affect at *t*-1 on rumination at *t*, ${log\psi}_{RU}$= log-transformed residual variance of rumination,$\log\psi_{NA}$= log-transformed residual variance of negative affect.

## Standardized Model Results

### Model 1

| Table S2  *Standardized Fixed Effects (median) of Model 1* | | | |
| --- | --- | --- | --- |
|  | *Estimate* | *95% CI* | |
| Parameters |  | *Lower* | *Upper* |
| *Autoregressive effects* | | | |
| $\phi_{RURU}$ | 0.204 | 0.177 | 0.232 |
| $\phi_{NANA}$ | 0.375 | 0.349 | 0.401 |
| *Effects of mindfulness intervention* | | | |
| $\beta_{MIRU}$ | -0.158 | -0.189 | -0.128 |
| $\beta_{MINA}$ | -0.114 | -0.143 | -0.085 |
| *Log residual variances* | | | |
| $\psi_{RU}$ | 0.812 | 0.790 | 0.835 |
| $\psi_{NA}$ | 0.740 | 0.715 | 0.764 |
| *Note.* Intercept estimates are not included in the standardized output; CI = credible interval, RU = rumination, NA = negative affect, MI = mindfulness intervention, $\phi_{RURU}$= autoregressive effect of rumination, $\phi_{NANA}$ = autoregressive effect of negative affect, $\beta_{MIRU}$ = effect of mindfulness intervention at *t* on rumination at *t*, $\beta_{MINA}$= effect of mindfulness intervention at *t* on negative affect at *t*, $\psi_{RU}$= log transformed residual variance of rumination,$\psi_{NA}$= log transformed residual variance of negative affect. | | | |

### Model 2

| Table S3  *Standardized Fixed Effects (median) of Model 2* | | | |
| --- | --- | --- | --- |
|  | *Estimate* | *95% CI* | |
| Parameters |  | *Lower* | *Upper* |
| *Autoregressive effects* | | | |
| $\phi_{RURU}$ | 0.205 | 0.176 | 0.232 |
| $\phi_{NANA}$ | 0.317 | 0.292 | 0.341 |
| *Cross-regressive effects* | | | |
| $\beta_{RUNA}$ | 0.268 | 0.236 | 0.297 |
| *(Interaction) Effects of mindfulness intervention* | | | |
| $\beta_{MINA}$ | -0.134 | -0.197 | -0.075 |
| $\beta_{MI*RUNA}$ | 0.082 | 0.026 | 0.143 |
| *Log residual variances* | | | |
| $\psi_{RU}$ | 0.920 | 0.907 | 0.934 |
| $\psi_{NA}$ | 0.605 | 0.570 | 0.635 |
| *Note.* Intercept estimates are not included in the standardized output; CI = credible interval, RU = rumination, NA = negative affect, MI = mindfulness intervention, $\phi_{RURU}$= autoregressive effect of rumination, $\phi_{NANA}$ = autoregressive effect of negative affect, $\beta_{RUNA}$= cross-regressive effect of rumination at *t* on negative affect at *t*, $\beta_{MINA}$ = effect of mindfulness intervention at *t* on negative affect at *t*, $\beta_{MI*RUNA}$= effect of the mindfulness intervention at *t* on the cross-regressive effect of rumination at *t* on negative affect at *t*, $\psi_{RU}$= log transformed residual variance of rumination,$\psi_{NA}$= log transformed residual variance of negative affect. | | | |

### Model 3

| Table S4  *Standardized Fixed Effects (median) of Model 3* | | | |
| --- | --- | --- | --- |
|  | *Estimate* | *95% CI* | |
| Parameters |  | *Lower* | *Upper* |
| *Autoregressive effects* | | | |
| $\phi_{RURU}$ | 0.155 | 0.124 | 0.184 |
| $\phi_{NANA}$ | 0.328 | 0.303 | 0.352 |
| *Cross-regressive effects* | | | |
| $\phi_{NARU}$ | 0.041 | 0.011 | 0.071 |
| *(Interaction) Effects of mindfulness intervention* | | | |
| $\beta_{MIRU}$ | -0.116 | -0.143 | -0.089 |
| $\beta_{MINA}$ | -0.064 | -0.091 | -0.038 |
| $\beta_{MI*NARU}$ | 0.014 | -0.014 | 0.044 |
| $\beta_{MI*RURU}$ | 0.004 | -0.027 | 0.031 |
| $\beta_{MI*NANA}$ | 0.002 | -0.026 | 0.030 |
| *Log residual variances* | | | |
| $\psi_{RU}$ | 0.829 | 0.812 | 0.847 |
| $\psi_{NA}$ | 0.810 | 0.792 | 0.828 |
| *Note.* Intercept estimates are not included in the standardized output; CI = credible interval, RU = rumination, NA = negative affect, MI = mindfulness intervention, $\phi_{RURU}$= autoregressive effect of rumination, $\phi_{NANA}$ = autoregressive effect of negative affect, $\phi_{NARU}$ = cross-regressive effect of negative affect at *t*-1 on rumination at *t*, $\beta_{MIRU}$= effect of mindfulness intervention at *t* on rumination at *t*, $\beta_{MINA}$= effect of mindfulness intervention at *t* on negative affect at *t*, $\beta_{MI*NARU}$ = effect of the mindfulness intervention at *t* on the cross-regressive effect of negative affect at *t*-1on rumination at *t*, $\beta_{MI*RURU}$ $\beta$*_MI*RURU_* = effect of the mindfulness intervention at *t* on the autoregressive effect of rumination, $\beta_{MI*NANA}$ = effect of the mindfulness intervention at *t* on the autoregressive effect of negative affect, $\psi_{RU}$= log transformed residual variance of rumination,$\psi_{NA}$= log transformed residual variance of negative affect. | | | |

# Manipulation Check

Figures S11-S13 include the latent decomposition, the within-person model, and the between-person model of the dynamics structural equation modeling (DSEM) analyses conducted as a manipulation check. For detailed model specification and results, see Mplus output files at <https://osf.io/y3gnt/> in the folder “Momentary Mindfulness and Rumination”/”Code and Results”/”3_Manipulation Check”.

Figure S11

*Latent Decomposition of DSEM Model Used as a Manipulation Check*


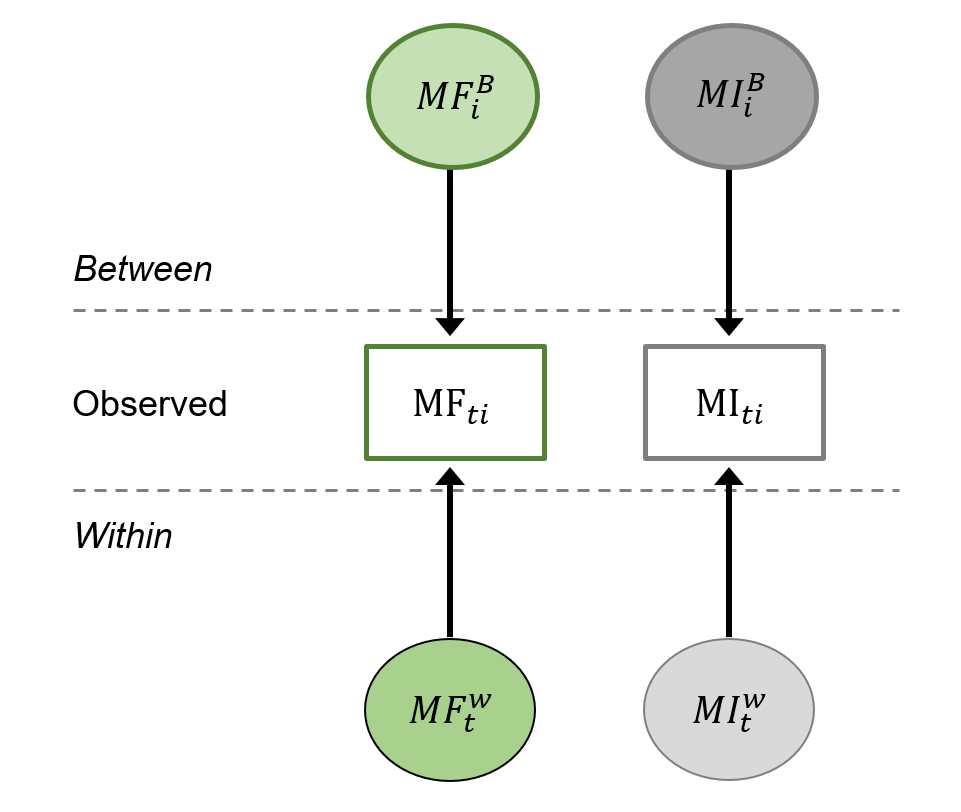


*Note*. MF: state mindfulness; MI = mindfulness intervention, *W* = within-person component of DSEM model, *B* = between-person component of DSEM model, *i* = individual *i*, *t* = occasion *t*

Figure S12

*Within-Person Model of DSEM Model Used as a Manipulation Check*


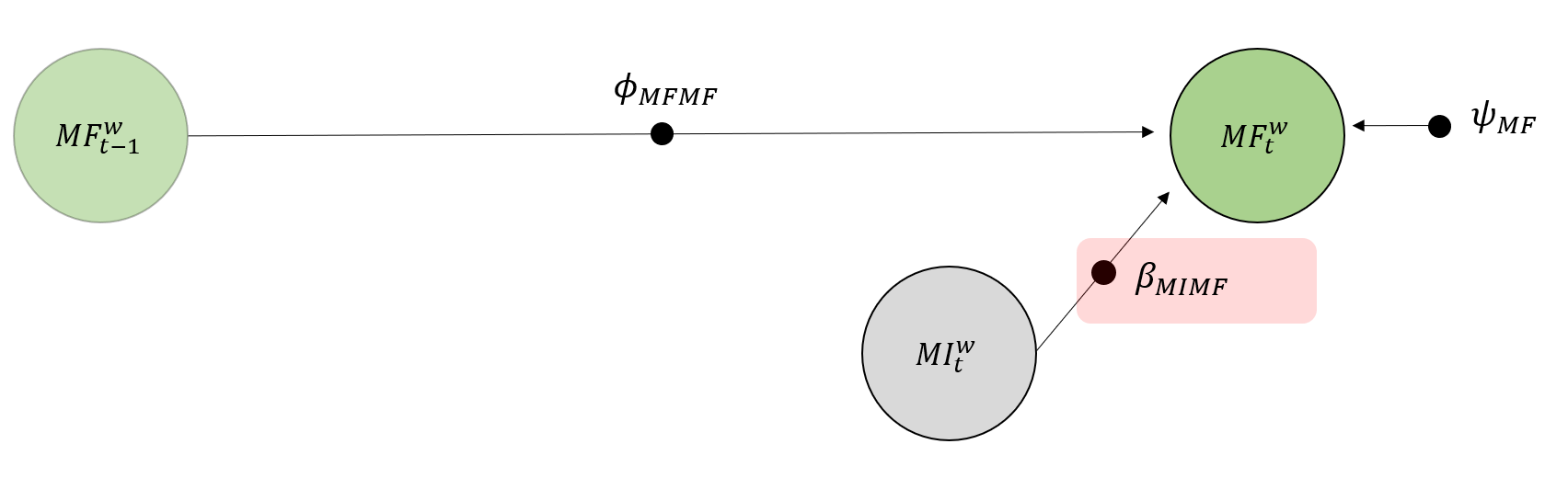


*Note*. MF: state mindfulness; MI = mindfulness intervention, *W* = within-person component of DSEM model, *t* = occasion *t*, $\phi_{MFMF}$ = autoregressive effect of state mindfulness, $\beta_{MIMF}$ = effect of mindfulness intervention at *t* on state mindfulness at *t*, $\psi_{MF}$= residual variance of state mindfulness. The filled circles indicate that the parameters are random.

Figure S13

*Between-Person Model of DSEM Model Used as a Manipulation Check*


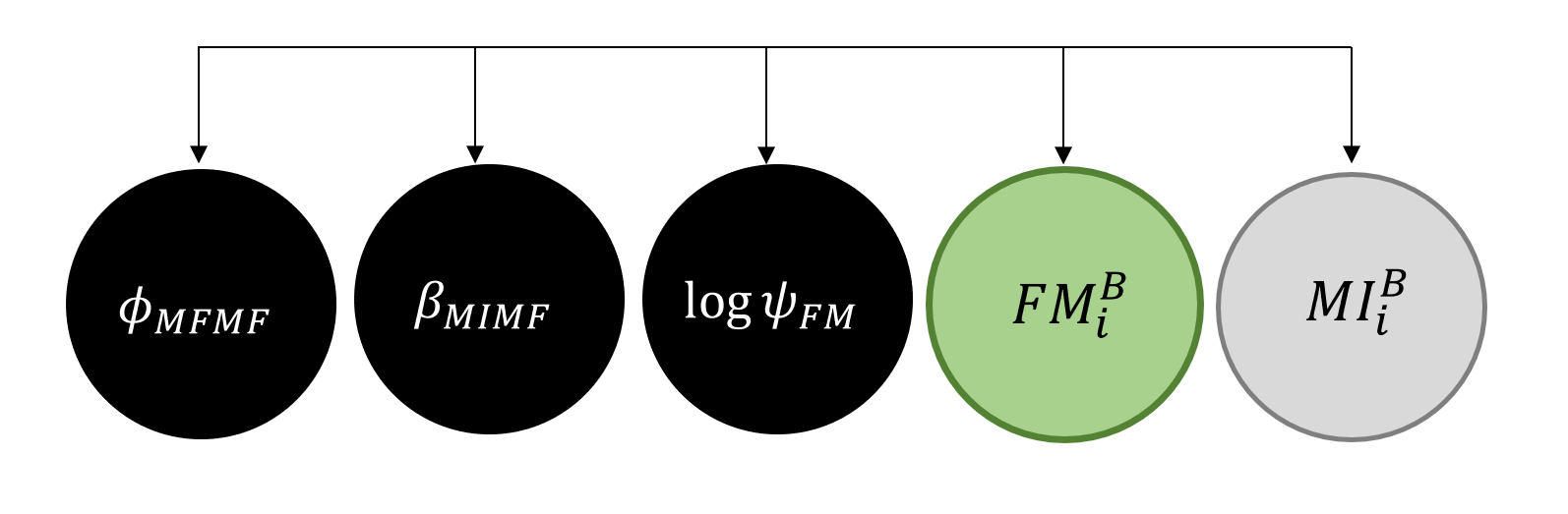


*Note*. MF: state mindfulness; MI = mindfulness intervention, *B* = between-person component of DSEM model, *i* = individual *i*, $\phi_{MFMF}$ = autoregressive effect of state mindfulness, $\beta_{MIMF}$ = effect of mindfulness intervention at *t* on state mindfulness at *t*, ${log\psi}_{MF}$= log transformed residual variance of state mindfulness.

# Supplemental Analyses

We ran the following supplementary analyses (SA) to check the robustness of our findings to alternate model specifications:

- Alternate approaches to dealing with unequal time intervals:
  - **SA (a)**
    Models 1 & 2: we repeated these models using a continuous time indicator (hours passed since last survey) as the TINTERVAL variable. For detailed model specification and results, please see Table S6 & S7 and Mplus output files at <https://osf.io/y3gnt/> in the folder “Momentary Mindfulness and Rumination”/”Code and Results”/”5_Supplemental Analyses”/”a TINTERVAL”;
  - **SA (b)**
    Model 3: we repeated this model after replacing the observed score on the first occasion on each new day in lagged variables with a missing value to exclude “overnight lags”. For detailed model specification and results, see Table S8 and Mplus output files at <https://osf.io/y3gnt/> in the folder “Momentary Mindfulness and Rumination”/”Code and Results”/”5_Supplemental Analyses”/”b lag”.
- Alternate approaches to dealing with intervention non-compliance:
  - **SA (c)**
    We repeated Models 1-3 including data from *n*=6 participants who were excluded for high (>40%) non-compliance with the intervention after the completion of data collection. This led to a sample size of *N*=97 in these analyses. Note that data from participants excluded for intervention non-compliance *prior* to completion of the study will not be analyzed. For detailed model specification and results, see Table S6-S8 and Mplus output files at <https://osf.io/y3gnt/> in the folder “Momentary Mindfulness and Rumination” /”Code and Results”/”5_Supplemental Analyses”/”c N=97”;
  - **SA (d)**We repeated Models 1-3 after removing all rows (ESM surveys) for which we had evidence of non-compliance with the intervention (i.e., where the total reaction time to the intervention/control prompt and the first 3 ESM items was <180000 ms). For detailed model specification and results, see Table S6-S8 and Mplus output files at <https://osf.io/y3gnt/> in the folder “Momentary Mindfulness and Rumination”/”Code and Results”/”5_Supplemental Analyses”/”d intervention non-compliance”.
- Including random residual covariance:
  - **SA (e)**We repeated Models 1-3 including a random residual covariance among the Within level outcomes (NA and RU). Following Hamaker et al. (2018), we fixed the factor loadings for the latent factor representing the residual covariance (and freely estimate its variance), which restricts the random residual covariance to take either a positive or negative value for all individuals. Given that negative affect (NA) and rumination (RU) are known to be positively correlated, we restricted the random residual covariance to positive values. For detailed model specification and results, see Table S6-S8 and Mplus output files at <https://osf.io/y3gnt/> in the folder “Momentary Mindfulness and Rumination”/”Code and Results”/”5_Supplemental Analyses”/”e random residual covariance”. Model 2 with covariance between the residual variances did not converge. Therefore, we do not report the results of this model in Table S7.

| **Table S5**  *Unstandardized Fixed Effects (median) from Model 1 for Supplementary Analyses* | | | | | | | |  |  |  |  |  |  |  |  |
| --- | --- | --- | --- | --- | --- | --- | --- | --- | --- | --- | --- | --- | --- | --- | --- |
|  | **(a)** with continuous time indicator (TINTERVAL) | | |  | **(c)** *N*=97*; n*=6 participants with high non-compliance included | | |  | **(d)** intervention non-compliant beeps removed | | |  | **(e)** random residual covariance included | | |
|  | *Estimate* | *95% CI* | |  | *Estimate* | *95% CI* | |  | *Estimate* | *95% CI* | |  | *Estimate* | *95% CI* | |
| Parameters |  | *Lower* | *Upper* |  |  | *Lower* | *Upper* |  |  | *Lower* | *Upper* |  |  | *Lower* | *Upper* |
| RU intercept | 36.030 | 32.322 | 39.701 |  | 35.954 | 32.453 | 39.368 |  | 35.931 | 32.213 | 39.732 |  | 36.008 | 32.365 | 39.679 |
| NA intercept | 15.880 | 13.540 | 18.288 |  | 16.197 | 13.884 | 18.468 |  | 15.856 | 13.472 | 18.236 |  | 15.930 | 13.532 | 18.308 |
| *Autoregressive effects* | | | |  |  |  |  |  |  |  |  |  |  |  |  |
| $\phi_{RURU}$ | 0.213 | 0.158 | 0.267 |  | 0.201 | 0.149 | 0.251 |  | 0.208 | 0.151 | 0.263 |  | 0.207 | 0.155 | 0.258 |
| $\phi_{NANA}$ | 0.399 | 0.342 | 0.455 |  | 0.377 | 0.321 | 0.432 |  | 0.382 | 0.322 | 0.440 |  | 0.362 | 0.306 | 0.418 |
| *Effects of mindfulness intervention* | | | |  |  |  |  |  |  |  |  |  |  |  |  |
| $\beta_{MIRU}$ | -2.691 | - 3.972 | - 1.410 |  | -2.653 | -3.829 | -1.495 |  | -2.872 | -4.124 | -1.612 |  | -2.198 | -3.335 | -1.048 |
| $\beta_{MINA}$ | -1.172 | - 1.690 | - 0.670 |  | -1.217 | -1.713 | -0.736 |  | -1.156 | -1.667 | -0.676 |  | -0.880 | -1.262 | -0.516 |
| *Log residual variances* | | | |  |  |  |  |  |  |  |  |  |  |  |  |
| $\psi_{RU}$ | 5.391 | 5.226 | 5.553 |  | 5.394 | 5.236 | 5.551 |  | 5.403 | 5.233 | 5.570 |  | 5.214 | 5.050 | 5.376 |
| $\psi_{NA}$ | 3.852 | 3.622 | 4.084 |  | 3.920 | 3.688 | 4.142 |  | 3.851 | 3.613 | 4.084 |  | 2.703 | 2.293 | 3.044 |
| *Note.* CI = credible interval, RU = rumination, NA = negative affect, MI = mindfulness intervention, $\phi_{RURU}$= autoregressive effect of rumination, $\phi_{NANA}$ = autoregressive effect of NA, $\beta_{MIRU}$ = effect of mindfulness intervention at *t* on rumination at *t*, $\beta_{MINA}$= effect of mindfulness intervention at *t* on NA at *t*, $\psi_{RU}$= log transformed residual variance of rumination,$\psi_{NA}$= log transformed residual variance of NA. | | | | | | | | | | | | | | | |

| **Table S6**  *Unstandardized Fixed Effects (median) from Model 2 for Supplementary Analyses* | | | | | | | | | | | |
| --- | --- | --- | --- | --- | --- | --- | --- | --- | --- | --- | --- |
|  | **(a)** with continuous time indicator (TINTERVAL) | | |  | **(c)** *N*=97*; n*=6 participants with high non-compliance included | | |  | **(d)** intervention non-compliant beeps removed | | |
|  | *Estimate* | *95% CI* | |  | *Estimate* | *95% CI* | |  | *Estimate* | *95% CI* | |
| Parameters |  | *Lower* | *Upper* |  |  | *Lower* | *Upper* |  |  | *Lower* | *Upper* |
| RU intercept | 35.936 | 32.250 | 39.597 |  | 35.899 | 32.439 | 39.411 |  | 35.860 | 32.157 | 39.514 |
| NA intercept | 16.736 | 13.798 | 19.719 |  | 16.238 | 13.885 | 18.604 |  | 15.930 | 13.550 | 18.349 |
| *Autoregressive effects* | | | |  |  |  |  |  |  |  |  |
| $\phi_{RURU}$ | 0.213 | 0.156 | 0.269 |  | 0.201 | 0.149 | 0.253 |  | 0.207 | 0.207 | 0.265 |
| $\phi_{NANA}$ | 0.327 | 0.272 | 0.381 |  | 0.321 | 0.268 | 0.375 |  | 0.322 | 0.266 | 0.379 |
| *Cross-regressive effects* | | | |  |  |  |  |  |  |  |  |
| $\beta_{RUNA}$ | 0.163 | 0.120 | 0.206 |  | 0.163 | 0.124 | 0.205 |  | 0.159 | 0.117 | 0.202 |
| *(Interaction) Effects of mindfulness intervention* | | | |  |  |  |  |  |  |  |  |
| $\beta_{MINA}$ | -1.062 | -1.694 | -0.494 |  | -1.139 | -1.739 | -0.543 |  | -1.167 | -1.808 | -0.544 |
| $\beta_{MI*RUNA}$ | 0.024 | -0.014 | 0.061 |  | 0.022 | -0.015 | 0.058 |  | 0.029 | -0.010 | 0.069 |
| *Log residual variances* | | | |  |  |  |  |  |  |  |  |
| $\psi_{RU}$ | 5.545 | 5.392 | 5.695 |  | 5.541 | 5.395 | 5.687 |  | 5.552 | 5.393 | 5.709 |
| $\psi_{NA}$ | 3.695 | 3.466 | 3.928 |  | 3.775 | 3.552 | 3.999 |  | 3.689 | 3.453 | 3.922 |
| *Note.* CI = credible interval, RU = rumination, NA = negative affect, MI = mindfulness intervention, $\phi_{RURU}$= autoregressive effect of rumination, $\phi_{NANA}$ = autoregressive effect of NA, $\beta_{RUNA}$= cross-regressive effect of rumination at *t* on NA at *t*, $\beta_{MINA}$ = effect of mindfulness intervention at *t* on NA at *t*, $\beta_{MI*RUNA}$= effect of the mindfulness intervention at *t* on the cross-regressive effect of rumination at *t* on NA at *t*, $\psi_{RU}$= log transformed residual variance of rumination,$\psi_{NA}$= log transformed residual variance of NA. | | | | | | | | | | | |

| **Table S7**  *Unstandardized Fixed Effects (median) of Model 3 for Supplementary Analyses* | | | | | | | |  |  |  |  |  |  |  |  |
| --- | --- | --- | --- | --- | --- | --- | --- | --- | --- | --- | --- | --- | --- | --- | --- |
|  | **(b)** “overnight lags” excluded | | |  | **(c)** *N*=97*; n*=6 participants with high non-compliance included | | |  | **(d)** intervention non-compliant beeps removed | | |  | **(e)** random residual covariance included | | |
|  | *Estimate* | *95% CI* | |  | *Estimate* | *95% CI* | |  | *Estimate* | *95% CI* | |  | *Estimate* | *95% CI* | |
| Parameters |  | *Lower* | *Upper* |  |  | *Lower* | *Upper* |  |  | *Lower* | *Upper* |  |  | *Lower* | *Upper* |
| RU intercept | 36.083 | 31.763 | 40.530 |  | 35.938 | 31.877 | 39.969 |  | 35.865 | 31.419 | 40.163 |  | 35.988 | 31.523 | 40.455 |
| NA intercept | 17.085 | 13.471 | 20.674 |  | 17.239 | 13.807 | 20.627 |  | 16.909 | 13.405 | 20.424 |  | 16.953 | 13.479 | 20.487 |
| *Autoregressive effects* | | | |  |  |  |  |  |  |  |  |  |  |  |  |
| $\phi_{RURU}$ | 0.163 | 0.106 | 0.220 |  | 0.150 | 0.097 | 0.200 |  | 0.157 | 0.100 | 0.214 |  | 0.155 | 0.104 | 0.207 |
| $\phi_{NANA}$ | 0.368 | 0.307 | 0.432 |  | 0.339 | 0.281 | 0.396 |  | 0.340 | 0.281 | 0.399 |  | 0.346 | 0.281 | 0.410 |
| *Cross-regressive effects* | | | |  |  |  |  |  |  |  |  |  |  |  |  |
| $\phi_{NARU}$ | 0.047 | -0.073 | 0.160 |  | 0.041 | -0.060 | 0.136 |  | 0.028 | -0.086 | 0.136 |  | 0.038 | -0.067 | 0.140 |
| *(Interaction) Effects of mindfulness intervention* | | | |  |  |  |  |  |  |  |  |  |  |  |  |
| $\beta_{MIRU}$ | -4.002 | -6.501 | -1.523 |  | -3.982 | -6.249 | -1.706 |  | -4.265 | -6.801 | -1.743 |  | -3.954 | -6.506 | -1.389 |
| $\beta_{MINA}$ | -1.396 | -2.310 | -0.519 |  | -1.562 | -2.445 | -0.704 |  | -1.454 | -2.403 | -0.529 |  | -1.593 | -2.606 | -0.646 |
| $\beta_{MI*NARU}$ | 0.054 | -0.145 | 0.257 |  | 0.035 | -0.136 | 0.211 |  | 0.041 | -0.144 | 0.234 |  | 0.044 | -0.133 | 0.218 |
| $\beta_{MI*RURU}$ | 0.018 | -0.086 | 0.120 |  | 0.018 | -0.072 | 0.106 |  | 0.018 | -0.086 | 0.127 |  | 0.015 | -0.080 | 0.110 |
| $\beta_{MI*NANA}$ | 0.009 | -0.088 | 0.105 |  | -0.011 | -0.099 | 0.079 |  | 0.017 | -0.079 | 0.113 |  | 0.014 | -0.074 | 0.104 |
| *Log residual variances* | | | |  |  |  |  |  |  |  |  |  |  |  |  |
| $\psi_{RU}$ | 5.445 | 5.258 | 5.633 |  | 5.433 | 5.254 | 5.615 |  | 5.442 | 5.246 | 5.640 |  | 5.179 | 4.952 | 5.397 |
| $\psi_{NA}$ | 3.926 | 3.655 | 4.199 |  | 3.965 | 3.703 | 4.222 |  | 3.913 | 3.643 | 4.189 |  | 2.004 | 1.164 | 2.647 |
| *Note.* CI = credible interval, RU = rumination, NA = negative affect, MI = mindfulness intervention, $\phi_{RURU}$= autoregressive effect of rumination, $\phi_{NANA}$ = autoregressive effect of NA, $\phi_{NARU}$ = cross-regressive effect of NA at *t*-1 on rumination at *t*, $\beta_{MIRU}$= effect of mindfulness intervention at *t* on rumination at *t*, $\beta_{MINA}$= effect of mindfulness intervention at *t* on NA at *t*, $\beta_{MI*NARU}$ = effect of the mindfulness intervention at *t* on the cross-regressive effect of NA at *t*-1on rumination at *t*, $\beta_{MI*RURU}$ $\beta$*_MI*RURU_* = effect of the mindfulness intervention at *t* on the autoregressive effect of rumination, $\beta_{MI*NANA}$ = effect of the mindfulness intervention at *t* on the autoregressive effect of NA, $\psi_{RU}$= log transformed residual variance of rumination,$\psi_{NA}$= log transformed residual variance of NA. | | | | | | | | | | | | | | | |

# Literature

Hamaker, E. L., Asparouhov, T., Brose, A., Schmiedek, F., & Muthén, B. (2018). At the Frontiers of Modeling Intensive Longitudinal Data: Dynamic Structural Equation Models for the Affective Measurements from the COGITO Study. *Multivariate Behavioral Research*, *53*(6), 820–841. https://doi.org/10.1080/00273171.2018.1446819
